# Human coronaviruses activate and hijack the host transcription factor HSF1 to enhance viral replication

**DOI:** 10.1007/s00018-024-05370-5

**Published:** 2024-09-07

**Authors:** Silvia Pauciullo, Anna Riccio, Silvia Santopolo, Anna Albecka, Guido Papa, Leo C. James, Sara Piacentini, Giulia Lanzilli, Antonio Rossi, M. Gabriella Santoro

**Affiliations:** 1 Department of Biology, University of Rome Tor Vergata, Rome, Italy; 2 MRC Laboratory of Molecular Biology, Francis Crick Avenue, Cambridge, CB2 0QH UK; 3 Institute of Translational Pharmacology, CNR, Rome, Italy

**Keywords:** HCoV-229E, HCoV-OC43, HCoV-NL63, Heat shock response, Protein homeostasis, SARS-CoV-2

## Abstract

Organisms respond to proteotoxic-stress by activating the heat-shock response, a cellular defense mechanism regulated by a family of heat-shock factors (HSFs); among six human HSFs, HSF1 acts as a proteostasis guardian regulating severe stress-driven transcriptional responses. Herein we show that human coronaviruses (HCoV), both low-pathogenic seasonal-HCoVs and highly-pathogenic SARS-CoV-2 variants, are potent inducers of HSF1, promoting HSF1 serine-326 phosphorylation and triggering a powerful and distinct HSF1-driven transcriptional-translational response in infected cells. Despite the coronavirus-mediated shut-down of the host translational machinery, selected HSF1-target gene products, including HSP70, HSPA6 and AIRAP, are highly expressed in HCoV-infected cells. Using silencing experiments and a direct HSF1 small-molecule inhibitor we show that, intriguingly, HCoV-mediated activation of the HSF1-pathway, rather than representing a host defense response to infection, is hijacked by the pathogen and is essential for efficient progeny particles production. The results open new scenarios for the search of innovative antiviral strategies against coronavirus infections.

**Supplementary Information:**

The online version contains supplementary material available at 10.1007/s00018-024-05370-5.

## Introduction

Protein homeostasis is essential for life in eukaryotes. Organisms respond to proteotoxic stress by activating a highly conserved cellular defense mechanism known as the heat-shock response (HSR) [1]. The HSR protects cells from the damaging effects of proteostasis disruption by different types of insults, including hyperthermia, by triggering the expression of cytoprotective heat-shock proteins (HSP) [2, 3]. HSPs, which include members of the HSP70 and HSP90 families, HSP27 and other proteins of the network, act as molecular chaperones that guide conformational states critical in the synthesis, folding, transport, assembly and degradation of proteins [2–4].

The HSR is regulated by a family of heat-shock (HS) transcription factors (HSFs) that are expressed and maintained in an inactive state under non-stress conditions. The human genome encodes six HSFs with different functions, among which HSF1 is the paralog responsible for regulating proteotoxic stress-driven transcriptional responses; HSF2 lacks intrinsic stress-responsiveness, but contributes to inducible HS genes expression through interplay with HSF1 [5–8].

HSF1 is a multi-domain transcription factor generally found as an inert monomer retained in the cytoplasm of unstressed cells in complex with several regulatory chaperones including the TRiC (TCP-1 ring-complex) nanomachine [9, 10]. Upon stress sensing, HSF1 is derepressed in a stepwise process that involves trimerization, nuclear translocation, phosphorylation/sumoylation and binding to DNA-sequences (heat-shock elements, HSE) characterized by inverted repeats of the pentameric motif -nGAAn- [6, 11, 12]. In human cells, beyond HS-genes, HSF1-binding sites have been described in a broad repertoire of genes encoding proteins with non-chaperone function [13–15].

Due to the abundant amount of viral proteins rapidly synthesized in bulk, several viruses are known to depend on the host chaperone machinery for correct protein folding and assembly into viral components during different phases of the virus replication cycle [16]. However, both a positive and a negative role of different HSP in the control of virus replication has been hypothesized and, in several cases, HSR activation was found to be detrimental to the invading pathogen [reviewed in 16, 17]. In particular, the role of HSR during coronavirus infection remains largely unknown.

**Fig. 1:**
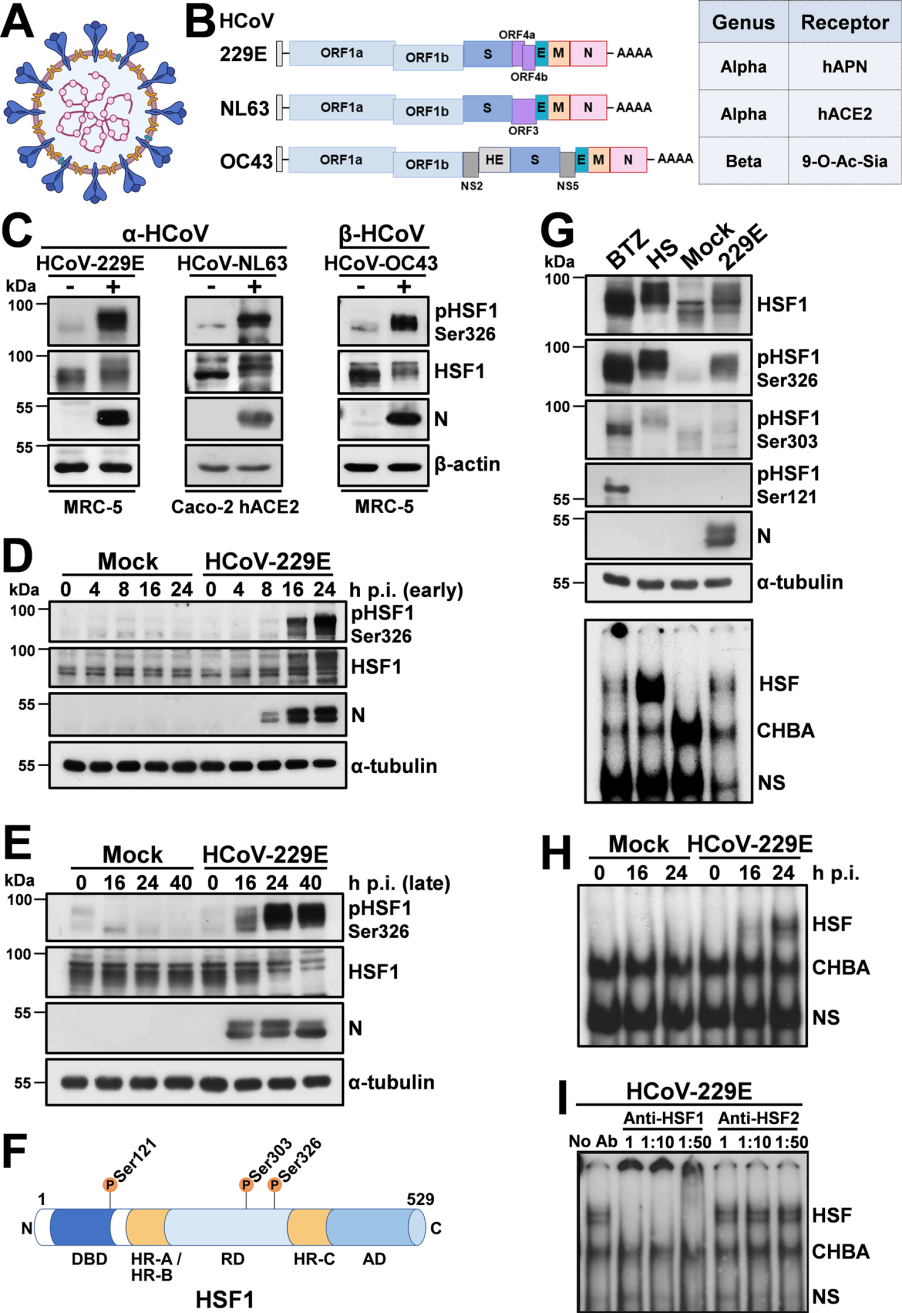
Human coronaviruses induce HSF1 phosphorylation and DNA-binding activity. **A** The human coronavirus lipid bilayer comprising the spike protein (blue), the membrane protein (orange) and the envelope protein (green), and the viral RNA (purple) associated with the nucleocapsid protein (pink) are shown. **B** Schematic representation of genome structure, classification and receptors of the human coronaviruses HCoV-229E, HCoV-NL63 and HCoV-OC43. ORF1a and ORF1b are represented as light blue boxes; genes encoding structural proteins spike (S), nucleocapsid (N), envelope (E), membrane (M), and hemagglutinin-esterase (HE) and genes encoding accessory proteins are shown. hAPN, human aminopeptidase N; 9-O-Ac-Sia, N-acetyl-9-O-acetylneuraminic acid; ACE2, angiotensin-converting enzyme 2. **C** Immunoblot (IB) analysis of pHSF1-Ser326, HSF1, viral nucleocapsid (N) and β-actin protein levels in MRC-5 and Caco-2 hACE2 cells mock-infected (−) or infected (+) with HCoV-229E or HCoV-OC43 (MRC-5) for 24 h, or HCoV-NL63 (Caco-2 hACE2) for 72 h at a m.o.i. of 0.1 TCID_50_/cell. **D**, **E** Whole-cell extracts (WCE) from samples mock-infected (Mock) or infected with HCoV-229E (1 TCID_50_/cell) were analyzed for pHSF1-Ser326, HSF1, N and α-tubulin protein levels at early (**D**) or late (**E**) times post infection (p.i.) by IB. **F** Schematic representation of HSF1 domain organization: DBD, DNA Binding Domain; HR-A/HR-B, Heptad Repeats A and B; RD, Regulatory Domain; HR-C, Heptad Repeat C; AD, Activation Domain. Phosphorylation sites Ser121, Ser303, and Ser326 are shown. **G** MRC-5 cells were treated with bortezomib (BTZ, 20 nM) for 16 h, exposed to heat stress (HS, 43 °C, 40 min), mock-infected (Mock) or infected with HCoV-229E (0.1 TCID_50_/cell) for 40 h. WCE were analyzed for levels of HSF1-Ser326, -Ser303 and -Ser121 phosphorylation, HSF1, viral N and α-tubulin proteins by IB (top panels). In the same samples, HSF1 DNA-binding activity was analyzed by EMSA (bottom panel). Positions of the HSF DNA-binding complex (HSF), constitutive HSE-binding activity (CHBA) and nonspecific protein-DNA interaction (NS) are shown. **H** MRC-5 cells were mock-infected or infected with HCoV-229E (1 TCID_50_/cell). At different times p.i., HSF1 DNA-binding activity was analyzed by EMSA. **I** WCE from samples infected with HCoV-229E (0.1 TCID_50_/cell, 40 h p.i.) were preincubated with different dilutions of anti-HSF1 or anti-HSF2 antibodies and analyzed by gel mobility supershift assay. The position of the nonsupershifted virus-induced HSF1 complex is indicated at the left (No Ab)

Coronaviruses (CoV) comprise a large number of enveloped, positive-sense single-stranded RNA viruses causing respiratory, enteric, renal and neurological diseases of varying severity in domestic and wild animals, as well as in humans [18]. Coronaviruses have the largest identified RNA genomes (typically 27-to-32 kb); all CoV genomes are arranged similarly with a large replicase-transcriptase gene encoded within the 5′-end preceding structural proteins encoded in the 3′-end, with an invariant gene order: 5′-S (spike)-E (envelope)-M (membrane)-N (nucleocapsid)-3′; numerous small open reading frames, encoding accessory proteins, are distributed among the structural genes (Fig. 1A, B). Some CoVs also encode an additional structural protein, hemagglutinin-esterase (HE) [19].

The genomic RNA complexes with the N protein to form a helical capsid structure surrounded by the viral envelope. Homotrimers of the class-I fusion glycoprotein S [20, 21] are embedded in the envelope and extend beyond the viral surface to bind to host receptors giving the virion its crown-like morphology (Fig. 1A). Other typical CoV features include: the expression of many nonstructural genes by ribosomal frameshifting, transcription of downstream genes by synthesis of 3′ nested sub-genomic mRNAs, and the presence of several unusual enzymatic activities encoded within the replicase-transcriptase polyprotein [22]. The unique replicative mechanism of CoV involves noncontiguous transcription of the genome, leading to a high rate of recombination, which may play a role in viral evolution and interspecies infections [23].

On the basis of their phylogenetic relationships and genomic structures, CoV are subdivided into four genera: *Alpha-*, *Beta-*, *Gamma-* and *Delta-coronavirus*. Human coronaviruses (HCoV) were discovered in the 1960s and were originally thought to cause only mild disease in humans [18, 19]. This view changed in the last 20 years with the emergence of highly-pathogenic SARS (Severe Acute Respiratory Syndrome) and MERS (Middle-East Respiratory Syndrome) coronaviruses [18, 19], and more recently with the devastating SARS-CoV-2 pandemic which caused over 775 million confirmed cases and 7 million deaths reported worldwide as of April 7, 2024 (https://covid19.who.int/).

Only two HCoV, HCoV-OC43 and HCoV-229E, were known prior to the emergence of SARS-CoV, while two more, HCoV-NL63 and HCoV-HKU1, were identified between 2004 and 2005 [24–27]. These four HCoV are globally distributed (seasonal CoV, sHCoV) and generally cause only mild upper respiratory diseases in immunocompetent hosts, although they can sometimes cause severe and even life-threatening infections especially in infants, elderly people, or immunocompromised patients [28–31]. Whereas all sHCoV cause respiratory tract infections, HCoV-OC43, HCoV-229E, HCoV-NL63 and HCoV-HKU1 are genetically dissimilar (Fig. 1B), belonging to two distinct taxonomic genera *(Alpha* and *Beta)*, and use different receptors: HCoV-229E and HCoV-NL63 have adopted cell surface enzymes as receptors, aminopeptidase-N (APN) for HCoV-229E and angiotensin-converting enzyme 2 (ACE2) for HCoV-NL63, while HCoV-OC43 and HCoV-HKU1 use 9-*O*-acetylated sialic acid [18, 32].

At present there is no information on HCoV impact on the host HSR. Herein we show that human coronaviruses trigger a remarkable and sustained activation of HSF1 in late stages of virus replication, leading to the expression of HSF1 canonical and non-canonical target genes in the infected cells. Interestingly, differently from other RNA viral pathogens [16, 17], the HSF1-directed transcriptional program turns out to be essential for an efficient virus replication cycle and progeny particles production.

## Results and discussion

### Human coronaviruses induce HSF1 phosphorylation, nuclear translocation and DNA-binding activity

During a study on the effect of proteostasis disruption in coronavirus-infected cells, we came across the unexpected finding that the human α-CoV 229E provoked the phosphorylation of HSF1 at serine-326 residue, which is considered crucial for HSF1 transcriptional activity [5, 33], in human lung cells (Fig. 1C). This accidental finding led us to investigate whether HCoV activate the HSR in infected cells. We first asked whether HSF1-phosphorylation is only triggered by HCoV-229E or is a common feature of human coronaviruses in different cell types. Human normal lung MRC-5 fibroblasts and colon carcinoma Caco-2 cells stably expressing the human ACE2-receptor (Caco-2 hACE2) were mock-infected or infected with α-HCoV-229E or β-HCoV-OC43 for 24 h and α-HCoV-NL63 (Caco-2 hACE2) for 72 h at a m.o.i. (multiplicity of infection) of 0.1 TCID_50_/cell. HCoV-HKU1 was not investigated because of its poor ability to grow in cell culture. At 24 h or 72 h post infection (p.i.), levels of HSF1, the phosphorylated form of HSF1 (pHSF1-Ser326) and the viral nucleocapsid protein were determined by immunoblot analysis. As shown in Fig. 1C, all three HCoVs trigger HSF1-Ser326 phosphorylation, as also indicated by slower migration of the factor on SDS–polyacrylamide gels.

To investigate the dynamic of HSF1-phosphorylation during coronavirus infection, MRC-5 cells were infected with HCoV-229E at different m.o.i. (0.1 or 1 TCID_50_/cell), and levels of HSF1-phosphorylation were analyzed at different times p.i.. Notably, high levels of HSF1-Ser326 phosphorylation were detected at late stages of the virus replication cycle, starting at 16 h p.i. and continuing up to 40 h p.i. (Fig. 1D, E, Supplementary Fig. S1A).

Human HSF1 can be post-translationally modified, including phosphorylation, sumoylation, ubiquitylation and acetylation, at > 50 residues (PhosphoSitePlus database: https://www.phosphosite.org). Among these, phosphorylation of the HSF1 Regulatory Domain (RD) residues, which is considered a hallmark of HSF1 activity/regulation, turns out to be very complex in human cells as phosphorylation of some sites, in particular Ser326, is pivotal for HSF1 activity, whereas at other sites (e.g., Ser303, in the RD; Ser121 in the DNA-Binding Domain) phosphorylation is associated with HSF1 activity attenuation [5] (Fig. 1F).

We therefore compared the effect of two major proteotoxic stressors, proteasome inhibition and heat-stress, to HCoV infection on the phosphorylation of different key HSF1 regulatory Ser-residues. MRC-5 cells were treated with the proteasome-inhibitor bortezomib (20 nM) for 16 h, or exposed to heat-stress (43 °C) for 40 min, or infected with HCoV-229E for 40 h. Whole-cell extracts were analyzed for levels of HSF1-phosphorylation at Ser326, Ser121 and Ser303 by immunoblot, and for HSF1 DNA-binding activity by EMSA. As expected, under the conditions utilized, heat-stress resulted in a remarkable increase in HSF1-Ser326 phosphorylation, causing a shift in HSF1 molecular weight, whereas only modestly affected Ser303 and had no effect on Ser121 phosphorylation at this time (Fig. 1G). Phosphorylation of all three serine residues was detected in cells exposed to bortezomib with Ser326 being predominant. Conversely, HCoV-infection selectively induced HSF1-Ser326 phosphorylation at levels comparable to heat-stress (Fig. 1G). In the same samples, Ser326 phosphorylation was associated with acquisition of HSF1 DNA-binding activity (Fig. 1G, bottom); concomitantly with HSF1-Ser326 phosphorylation, HSF1 DNA-binding activity was detected at late stages of the virus replication cycle, starting at 16 h p.i. (Fig. 1H). Similarly to heat-shock [12, 14], HSF1 was found to be the primary component of the virus-induced HSE-binding activity in HCoV-infected cells as determined by gel-mobility supershift assay, while HSF2 was not affected (Fig. 1I). Of note, differently from HCoV, infection with the rhabdovirus VSV (Vesicular Stomatitis virus) did not induce HSF1-phosphorylation independently of the m.o.i., indicating that HSF1 activation is not a general response to RNA-virus infection (Supplementary Fig. S1B).

**Fig. 2:**
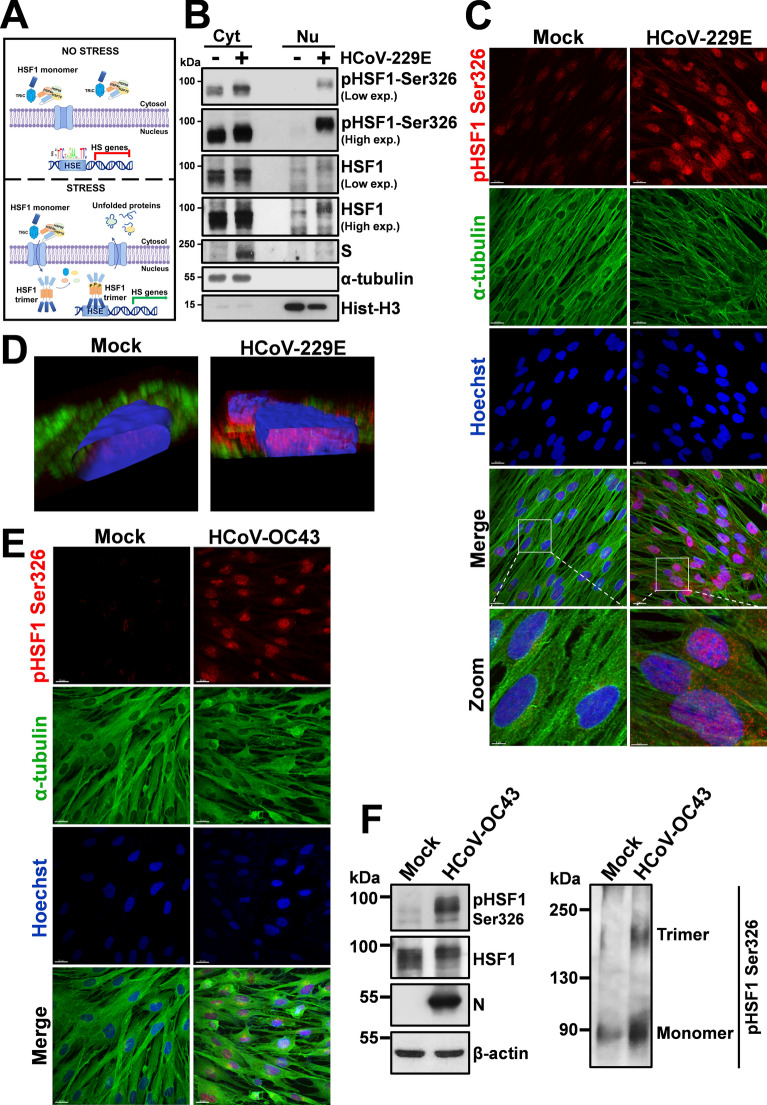
HCoV infection triggers HSF1 nuclear translocation in human lung cells. **A** Schematic representation of HSF1 intracellular localization under physiological (no stress) and stress conditions. **B** Immunoblot analysis of pHSF1-Ser326, HSF1 and viral spike (S) protein levels in cytoplasmic (Cyt) and nuclear (Nu) fractions of MRC-5 cells mock-infected (−) or infected (+) with HCoV-229E (0.1 TCID_50_/cell) for 24 h. Antibodies against α-tubulin and histone H3 (Hist-H3) were used as loading controls for cytoplasmic and nuclear fractions, respectively. **C** Confocal images of pHSF1-Ser326 (red) and α-tubulin (green) intracellular localization in MRC-5 cells mock-infected or infected with HCoV-229E (1 TCID_50_/cell) at 30 h p.i.. Nuclei are stained with Hoechst (blue). Merge and zoom images are shown. Scale bar, 20 μm (zoom, 5 μm). **D** Confocal 3D-reconstruction of pHSF1-Ser326 (red) intranuclear localization in MRC-5 cells mock-infected or infected as in C; α-tubulin is shown in green. Nuclei are stained with Hoechst (blue). The overlay of the fluorochromes is shown. **E** Confocal images of pHSF1-Ser326 (red) and α-tubulin (green) intracellular localization in MRC-5 cells mock-infected or infected with HCoV-OC43 (1 TCID_50_/cell) at 30 h p.i.. Nuclei are stained with Hoechst (blue). Merge images are shown. Scale bar, 20 μm. **F** IB of pHSF1-Ser326, HSF1, N and β-actin protein levels in MRC-5 cells mock-infected or infected with HCoV-OC43 (0.1 TCID_50_/cell) for 24 h (left panels). HSF1 monomers and trimers in the same samples are shown (right panel)

As described in the Introduction, upon stress-sensing HSF1 is derepressed in a stepwise process involving, beside post-translational modifications, trimerization and nuclear translocation (Fig. 2A). HSF1 intracellular localization was therefore determined by cell-fractionation and confocal-microscopy studies in MRC-5 cells infected with the α-HCoV-229E. As shown in Fig. 2B, C, under normal conditions HSF1 is predominantly localized in the cytoplasm of MRC-5 cells; HCoV-229E infection, in addition to Ser326-phosphorylation, caused a dramatic redistribution of the factor, which was found in the nuclei of infected cells in a remarkably abundant amount (Fig. 2C, D). Similar results were obtained in MRC-5 cells infected with the β-HCoV-OC43 that caused HSF1 Ser326-phosphorylation, trimerization and translocation to the nucleus (Fig. 2E, F). These results demonstrate that both *Alpha-* and *Beta*-coronaviruses trigger HSF1 phosphorylation and nuclear translocation.

### HCoV infection turns on a distinct HSF1-driven transcriptional program in human lung cells

**Fig. 3:**
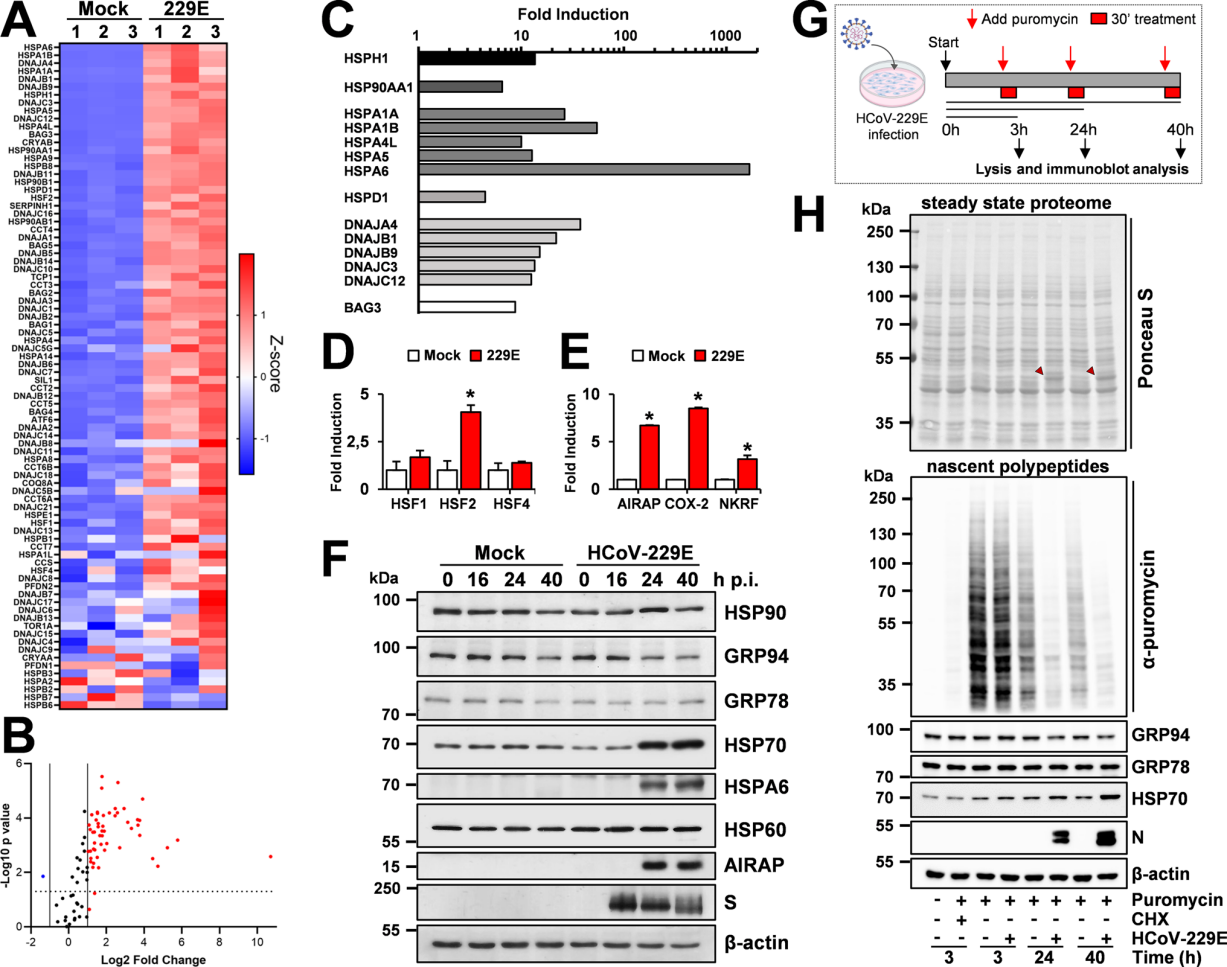
HCoV infection turns on an HSF1-driven transcriptional program in human lung cells. **A**–**D** Expression profile of selected HSF1-target genes affected by HCoV-229E infection (0.1 TCID_50_/cell) for 24 h in MRC-5 cells relative to mock-infected cells as determined by qRT-PCR array (PAHS-076ZD-2-Qiagen). Heat Map (**A**) and Volcano plot (**B**) of 84 human HSPs and chaperones/cochaperones gene expression. In **A** each row represents a single gene, each column represents a sample [mock-infected or HCoV-229E infected cells (229E); n = 3]. The gradual color ranging from blue to red represents the mRNA expression level (Z-score). In the Volcano plot (**B**) fold regulation threshold is set to 2 and *p*-value cut off is 0.05; each dot represents a gene: red dots indicate significantly upregulated genes and blue dots indicate significantly downregulated genes. Selected HSPs and chaperones/cochaperones genes whose expression is highly induced by HCoV infection are shown in **C**; levels of heat shock factors (HSF1, HSF2 and HSF4) gene expression affected by HCoV infection are shown in **D**. **E** Expression of non-canonical HSF1-target genes AIRAP, COX-2 and NKRF in samples treated as in A as determined by qRT-PCR. Error bars indicate means ± S.D. (n = 3). *p < 0.05; Student’s *t*-test (**D**, **E**). **F** Levels of HSP90, GRP94, GRP78, HSP70, HSPA6, HSP60, AIRAP, viral spike (S) and β-actin proteins were determined by IB in MRC-5 cells mock-infected or infected with HCoV-229E (1 TCID_50_/cell) at different times p.i.. **G** Schematic representation of the puromycin-labeling experimental protocol. **H** MRC-5 cells were mock-infected (−) or infected with HCoV-229E (+) (1 TCID_50_/cell), or treated with vehicle (−) or cycloheximide (CHX, 100 µg/ml, +) for 3 h, as positive control of translation inhibition. At different times p.i., puromycin (2.5 µg/ml, +) was added thirty minutes before harvesting and IB analysis. Blot membranes were stained with Ponceau S solution to assess the steady-state proteomes (top panel) and then hybridized with anti-puromycin antibodies to detect de novo synthesized nascent polypeptides (middle panel). HCoV-N protein is indicated by red arrowheads. Levels of GRP94, GRP78, HSP70, HCoV-N and β-actin proteins detected by IB in the same samples are shown (bottom panels)

Since phosphorylation, nuclear translocation and DNA-binding activity in some instances may not warrant target-genes transcription [34, 35], we next investigated whether HCoV-induced nuclear HSF1 is transcriptionally active. HSF1-target gene expression was compared in MRC-5 cells mock-infected or infected with HCoV-229E for 24 h using a qRT-PCR array, which profiles the expression of 84 HS-genes encoding HSPs and molecular chaperones. HCoV infection resulted in the expression of high levels of several HS-genes (Fig. 3A, B, Supplementary Table 1), demonstrating that virus-induced HSF1 is transcriptionally active. Notably, the HCoV-induced HSF1 transcriptional program appears to differ from the HSF1-dependent gene expression profile induced by heat-stress (43 °C, 40 min) in MRC-5 cells (Supplementary Table 2, Supplementary Fig. S2).

Whereas in the classical heat-shock response HSF1 was generally considered to function as a guardian of cellular health, accumulating evidence have challenged these traditional views, and HSF1 was shown to drive diverse transcriptional programs in development, metabolism and cancer that are distinct from the classical HSR [36–38]. In the case of the HCoV-driven response we found that viral infection resulted in the expression of high levels of different HSP mRNAs belonging to the HSP70 and HSP90 families, as well as glucose-regulated proteins GRP78/BiP (HSPA5), GRP94 (HSP90B1) and a plethora of other HSF1-target genes. The expression of DNAJ (HSP40) family chaperones, characterized by a highly conserved amino acid stretch (J-domain) and with critical functions in protein folding and assembly, is also notably increased; in particular, DNAJA4 expression was increased more than 35-fold, and DNAJB1 (Hdj1/Sis1), DNAJB9 (ERdj4), DNAJC3 (ERdj6) and DNAJC12 more than tenfold (Fig. 3C). HSPH1 gene (HSP110-family member) expression was also elevated, whereas genes belonging to the HSP60 (HSPD1) family were increased to a lesser extent (Fig. 3A, C). Extremely high levels were detected for the HSPA6 gene product, whose expression was increased more than 1600-fold; notably, HCoV infection also strongly (> eightfold) increased the expression of the HSP70 co-chaperone BAG3 (Bcl-2-associated athanogene domain-3), a multidomain protein with anti-apoptotic function playing a central role in autophagy and involved in the dynein motor pathway regulation (Fig. 3C) [39, 40].

Furthermore, whereas HCoV infection did not affect HSF1 or HSF4 expression, it resulted in a significant increase of HSF2 expression (Fig. 3A, D), confirming the recent observation that HSF1 promotes HSF2 gene transcription in human cells [41].

As indicated above, beyond HS genes, HSF1-binding sites were described in several genes encoding proteins with non-chaperone function [38, 42]. We have previously identified three human non-canonical HSF1-target genes: (1) AIRAP (arsenite-inducible RNA-associated protein) [8, 14], recently identified as a regulator of prosurvival networks in melanoma cells [43]; (2) NKRF (NF-κB repressing factor), known as a silencer of the pro-inflammatory mediator NF-κB [44, 45], recently shown to be crucial for correct ribosomal-RNA processing and preventing aberrant rRNA-precursors accumulation [15]; (3) cyclooxygenase-2 (hCOX-2), a key regulator of inflammation, which is temperature-regulated in human cells via a distal *cis*-acting HSE [12]. Interestingly, the expression of AIRAP, NKRF and COX-2 was significantly increased in HCoV-229E-infected MRC-5 cells (Fig. 3E). Notably, the expression of HSF1-target genes was detected up to 40 h after infection and was dependent on the virus m.o.i. (Supplementary Fig. S1C, D).

Similarly to many RNA viruses, HCoVs affect the host-cell translational machinery turning off cellular protein synthesis to promote viral RNA translation employing, in addition to cap-dependent translation, non-canonical translation mechanisms to expand their coding capacity such as ribosomal frameshifting and ribosomal shunting [22, 46]; therefore, induction of cellular genes expression, even at high levels, not necessarily may lead to an increase in the relative protein levels. Analysis of a set of canonical and non-canonical HSP indicated that the level of some, but not all, proteins examined was increased in HCoV-infected cells despite the virus-induced severe shut-down of host protein synthesis detected by puromycin-labeling experiments; in particular, HSP70, HSPA6 and AIRAP levels were remarkably high in HCoV-229E-infected MRC-5 cells at late stages of infection, whereas no significant changes in the levels of HSP90, HSP60, GRP78 and GRP94 were detected at all times examined (Fig. 3F–H and Supplementary Fig. S3). Similarly to HCoV-229E, infection with HCoV-OC43 and HCoV-NL63 selectively triggered the expression of high levels of selected HSPs, independently of the host-cell type (Supplementary Fig. S4).

**Fig. 4:**
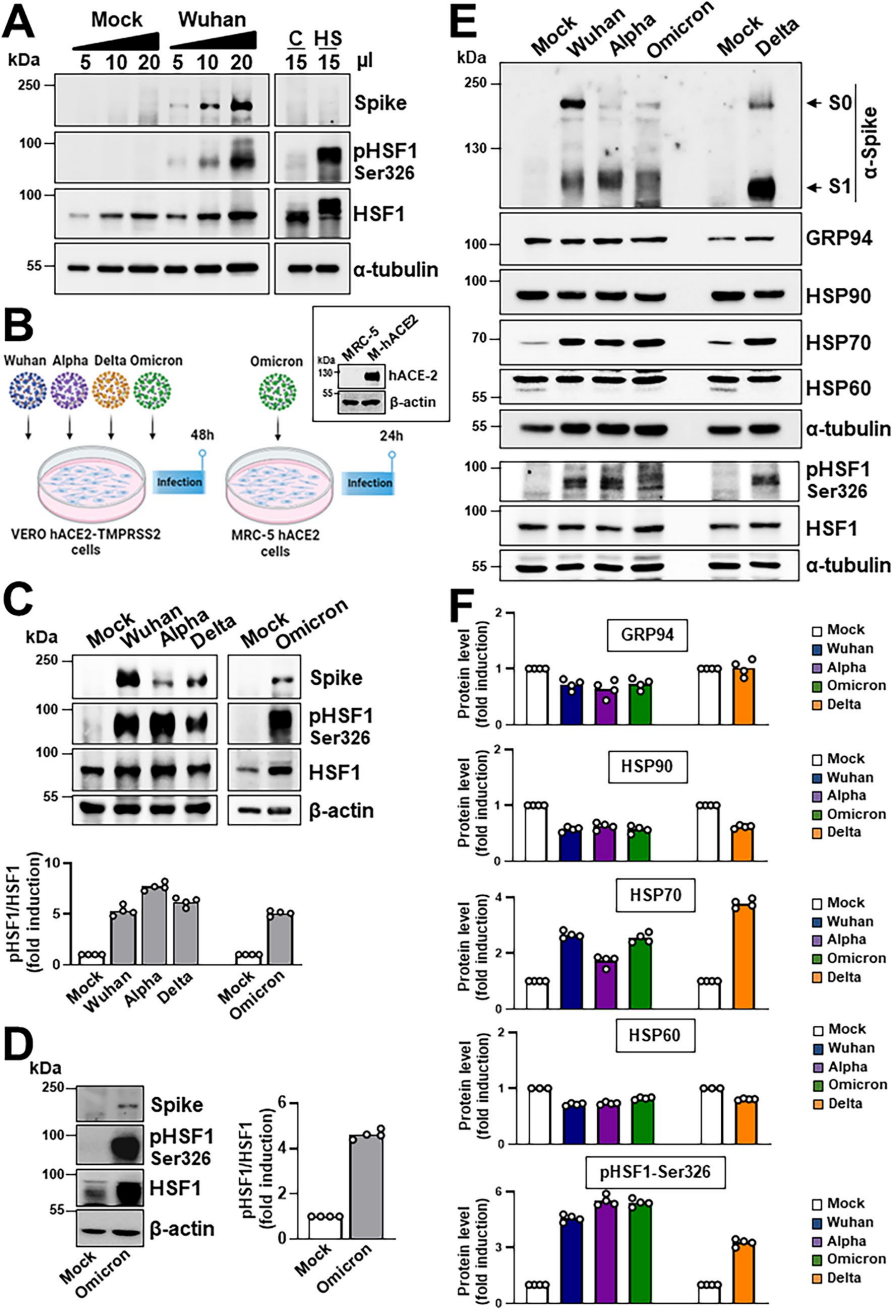
Effect of different SARS-CoV-2 variants on HSF1 activation and HSP70 expression in the host cell. **A** Immunoblot analysis of SARS-CoV-2 viral spike, pHSF1-Ser326, HSF1 and α-tubulin protein levels in Vero-hACE2-TMPRSS2 cells mock-infected or infected for 48 h with SARS-CoV-2 Wuhan strain (0.1 PFU/cell), or in Vero E6 cells exposed to heat-stress (HS, 43 °C, 40 min). **B** Schematic representation of the experimental design of SARS-CoV-2 variants infection of Vero-hACE2-TMPRSS2 or MRC5-hACE2 cells. Immunoblot analysis of human angiotensin-converting enzyme 2 (hACE2) protein levels in MRC-5 wild type or expressing the human ACE2-receptor (M-hACE2) is shown. **C** Vero-hACE2-TMPRSS2 cells were mock-infected or infected with Wuhan, Alpha, Delta (0.1 PFU/cell) or Omicron (0.5 PFU/cell) SARS-CoV-2 variants for 48 h. Equal amounts of whole-cell extracts (10 μl) were analyzed for levels of spike, HSF1-Ser326, HSF1 and β-actin by IB (top panels). The pHSF1/HSF1 ratio was determined after normalizing to β-actin and expressed as fold induction of the mock-infected control, which was arbitrarily set to 1 (bottom panel). **D** MRC5-hACE2 cells were mock-infected or infected with the SARS-CoV-2 Omicron BA.1 variant (0.1 PFU/cell) for 24 h. Equal amounts of whole-cell extracts (15 μl) were analyzed for levels of spike, HSF1-Ser326, HSF1 and β-actin (left panels). The pHSF1/HSF1 ratio was determined after normalizing to β-actin and expressed as fold induction of the mock-infected control, which was arbitrarily set to 1 (right panel). **E**, **F** Vero-hACE2-TMPRSS2 cells were mock-infected or infected with different SARS-CoV-2 variants as in **C**. Equal amounts of whole-cell extracts (20 μl) were analyzed for levels of spike, GRP94, HSP90, HSP70, HSP60, pHSF1-Ser326, HSF1 and α-tubulin by IB (**E**). Uncleaved S proteins (S0) and S1 subunits are indicated by arrows. Relative amounts of GRP94, HSP90, HSP70, HSP60 and pHSF1 were determined after normalizing to α-tubulin and expressed as fold induction of the mock-infected control, which was arbitrarily set to 1 (**F**). **C**, **D**, **F** *n* = 4 (two technical replicates over two biological replicates)

To investigate whether also highly-pathogenic HCoVs induce HSF1 activation triggering HSP expression, Vero cells expressing the human ACE2-receptor (hACE2) and transmembrane-protease serine-2 (Vero-hACE2-TMPRSS2) were infected with SARS-CoV-2 Wuhan strain (0.1 PFU/cell) for 48 h and levels of HSF1 phosphorylation were analyzed. As shown in Fig. 4A, SARS-CoV-2 infection was found to cause HSF1-Ser326 phosphorylation. Next, the ability of different SARS-CoV-2 variants, including Alpha, Delta and Omicron (BA.1), to induce HSF1 phosphorylation and HSP expression was compared. All variants were found to trigger HSF1 phosphorylation and selectively induce HSP70 expression (Fig. 4B–E). To investigate whether SARS-CoV-2 infection triggers HSF1 activation in human lung cells, MRC-5 cells, which are highly susceptible to HCoV-OC43 and HCoV-229E, but not to SARS-CoV-2 infection [47, 48], were engineered to stably express hACE2 (MRC5-hACE2 cells). Given the high levels of the hACE2 receptor (Fig. 4B) on their surface, MRC5-hACE2 cells were highly susceptible to SARS-CoV-2 infection. MRC5-hACE2 cells were infected with SARS-CoV-2 (Omicron BA.1, 0.1 PFU/cell) and levels of HSF1 phosphorylation were analyzed at 24 h p.i.. The results, shown in Fig. 4D, indicate that SARS-CoV-2 infection is able to induce HSF1 activation also in human lung cells. It should be noted that, in addition to Omicron BA.1, also the Wuhan, Alpha and Delta SARS-CoV-2 variants were observed to induce HSF1 phosphorylation in MRC5-hACE2 cells (Supplementary Fig. S5).

Taken together, these results demonstrate that HCoV trigger a powerful and distinct HSF1-driven transcriptional/translational response in infected cells at late stages of infection and prompted us to investigate whether this phenomenon only reflects a cellular defense response to the invading pathogen, or whether the virus activates and hijacks the HSF1-pathway for its own gain.

### HSF1 activation is required for efficient replication of human coronaviruses

Since HSR activation was shown to be detrimental for virus replication and to protect host cells from virus-induced damage during infection with several viruses belonging to different RNA virus families, including *Paramyxoviridae, Rhabdoviridae* and *Picornaviridae* [reviewed in 16, 17], we hypothesized that blocking the HCoV-induced HSF1 signaling would result in enhancing virus replication.

**Fig. 5:**
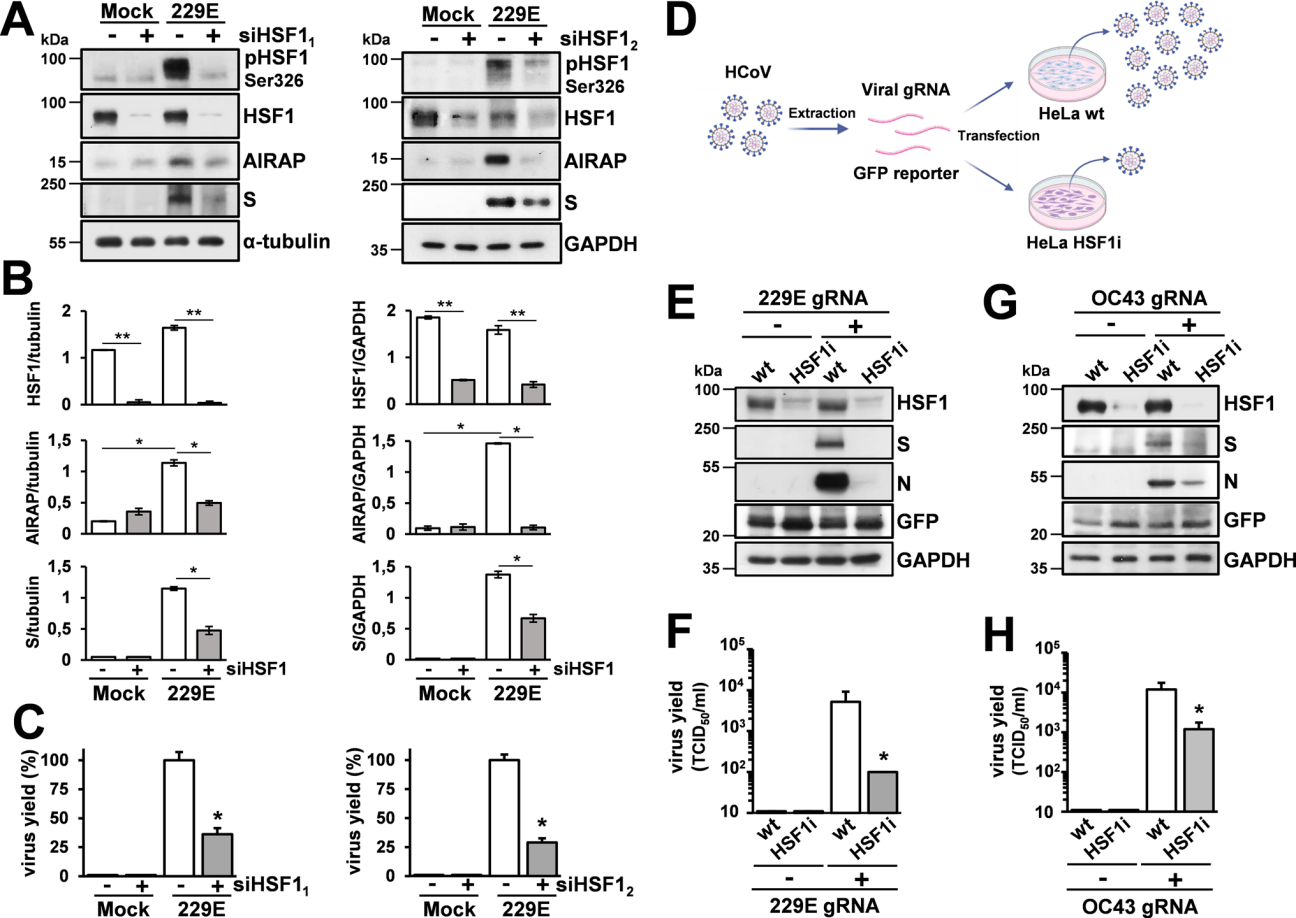
HSF1 is required for efficient HCoV replication. **A** Immunoblot of pHSF1-Ser326, HSF1, AIRAP, viral spike (S), α-tubulin and GAPDH protein levels in MRC-5 cells transiently transfected with two different HSF1-siRNAs [siHSF1_1_ (left) and siHSF1_2_ (right); +] or scramble-RNA (−) for 48 h, and infected with HCoV-229E (0.1 TCID_50_/cell) or mock infected (Mock) for 24 h. **B** The relative amount of total HSF1, AIRAP and viral S protein, normalized to the loading control in the same sample, were determined by densitometric analysis using ImageJ software. Error bars indicate means ± S.D. (n = 3). **C** In parallel, virus yield from supernatants of infected cells was determined at 24 h p.i. by TCID_50_ infectivity assay. Data, expressed as percentage of control, represent the mean ± S.D. (n = 3). *p < 0.05, **p < 0.01; Student’s *t*-test (**B**, **C**). **D** Schematic representation of HCoV genomic RNA transfection assays. **E–H** Wild-type (wt) or stably HSF1-silenced (HSF1i) HeLa cells were co-transfected with HCoV-229E (229E gRNA) or HCoV-OC43 (OC43 gRNA) genomic RNA and the pCMV-GFP vector for 4 h. After 48 h (229E) or 72 h (OC43), levels of HSF1, viral spike (S) and nucleocapsid (N), GFP and GAPDH proteins were analyzed by IB (**E**, **G**). In parallel, virus yield in the supernatant of transfected cells was determined by TCID_50_ infectivity assay (**F**, **H**). Data, expressed as TCID_50_/ml, represent the mean ± S.D. (n = 3). *p < 0.05; Student’s *t*-test

We first investigated the effect of HSF1-silencing on HCoV-229E infection. In a first set of experiments MRC-5 cells were transiently transfected with two different HSF1 siRNA (siHSF1_1_ and siHSF1_2_) or scramble-RNA and, after 48 h, were infected with HCoV-229E. An efficient HSF1-silencing was obtained with both HSF1-siRNAs, as confirmed also by lower AIRAP levels in HSF1-silenced infected cells (Fig. 5A, B). Interestingly, HSF1-silencing did not enhance virus replication, but instead resulted in decreasing both viral spike levels and viral progeny production at 24 h p.i. (Fig. 5A–C), suggesting that HSF1 may be necessary for optimal virus replication.

Next, we analyzed HCoV replication in stably HSF1-silenced HeLa cells (HeLa-HSF1i) as compared to wild-type (HeLa-WT) cells [49]. Because of the lack of appropriate receptors for α- and β-CoVs in these cells, 229E and OC43 HCoV genomic-RNA was extracted from infectious virions and transfected into HeLa-HSF1i or HeLa-WT monolayers together with a GFP-reporter plasmid (Fig. 5D). As shown in Fig. 5E–H, genomic-RNA transfection resulted in the production of infectious viral progeny in HeLa cells at 48 h or 72 h after transfection. Notably, both HCoV-229E and HCoV-OC43 virus yield and viral structural protein levels were significantly lower in HeLa-HSF1i cells as compared to wild-type cells (Fig. 5E–H), confirming that HSF1 is needed for both α- and β-HCoV efficient replication and indicating that HSF1 inhibitors may counteract coronavirus infection.

**Fig. 6:**
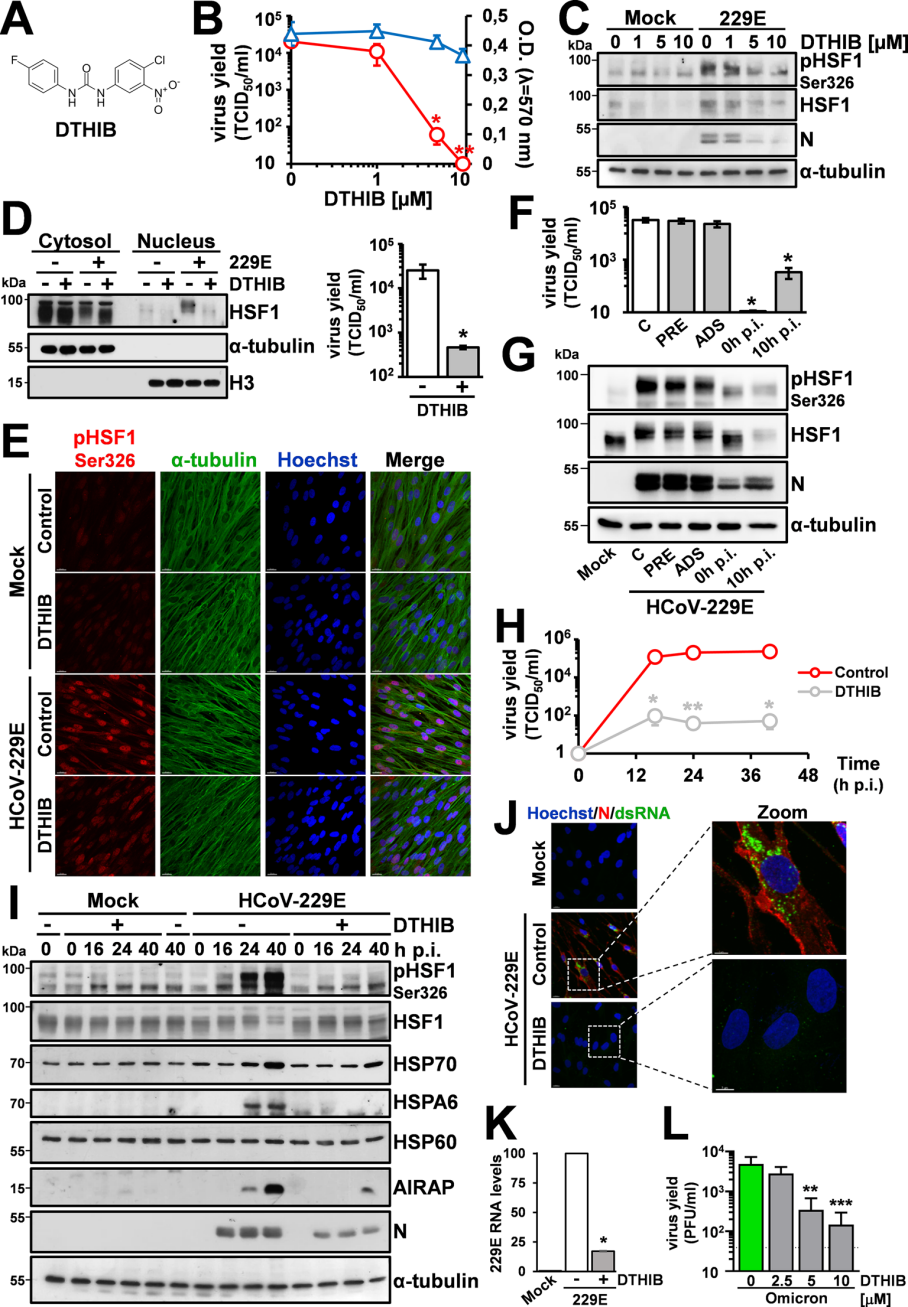
Inhibition of virus-induced HSF1 activation by DTHIB impairs HCoV replication. **A** Structure of DTHIB (Direct Targeted HSF1 Inhibitor). **B** MRC-5 cells mock-infected or infected with HCoV-229E (0.1 TCID_50_/cell) were treated with different concentrations of DTHIB immediately after the adsorption period. Virus yield (Ο) was determined at 24 h p.i. by TCID_50_ infectivity assay. Data, expressed as TCID_50_/ml, represent the mean ± S.D. (n = 4). *p < 0.05, **p < 0.01; ANOVA test. In parallel, cell viability (△) was determined in mock-infected cells by MTT assay. Absorbance (O.D.) of converted dye was measured at λ = 570 nm. **C** Immunoblot of pHSF1-Ser326, HSF1, viral N and α-tubulin protein levels in samples treated as in B. **D** Immunoblot analysis of HSF1 levels in cytoplasmic (Cytosol) and nuclear (Nucleus) fractions of MRC-5 cells mock-infected (−) or infected ( +) with HCoV-229E (0.1 TCID_50_/cell) for 24 h and treated with DTHIB (10 µM, +) or vehicle (−) immediately after the adsorption period (left panels). Antibodies against α-tubulin and Histone H3 (H3) were used as a loading control for cytoplasmic and nuclear fractions, respectively. Virus yield was determined at 24 h p.i. by TCID_50_ infectivity assay (right panel). Data, expressed as TCID_50_/ml, represent the mean ± S.D. (n = 4). *p < 0.05; Student’s *t*-test. **E** Confocal images of pHSF1-Ser326 (red) and α-tubulin (green) intracellular localization in MRC-5 cells mock-infected or infected with HCoV-229E (1 TCID_50_/cell) for 30 h and treated with DTHIB (5 µM) or control diluent after the adsorption period. Nuclei are stained with Hoechst (blue). Merge images are shown. Scale bar, 20 µm. **F** MRC-5 cells mock-infected or infected with HCoV-229E (0.1 TCID_50_/cell) were treated with 10 μM DTHIB (filled bars) or control vehicle (**C**, empty bar) at 3 h before infection (PRE), immediately after the adsorption period (0 h), at 10 h after infection (10 h p.i.), or only during the adsorption period (ADS). Virus yield was determined at 24 h p.i. by TCID_50_ assay. Data, expressed as TCID_50_/ml, represent the mean ± S.D. (n = 3). *p < 0.05; ANOVA test. **G** Immunoblot of pHSF1-Ser326, HSF1, AIRAP, HCoV-N and α-tubulin protein levels of samples treated as in **F**. **H**, **I** MRC-5 cells were mock-infected or infected with HCoV-229E (1 TCID_50_/cell) and treated with DTHIB (7.5 µM). At different times p.i., virus yield was determined by TCID_50_ infectivity assay. Data, expressed as TCID_50_/ml, represent the mean ± S.D. (n = 3). *p < 0.05, **p < 0.01; Student’s *t*-test (H). In parallel, levels of pHSF1-Ser326, HSF1, HSP70, HSPA6, HSP60, AIRAP, HCoV-N and α-tubulin proteins were determined by IB (**I**). **J** Confocal images of viral nucleoprotein (red) and dsRNA (green) intracellular localization in MRC-5 cells infected with HCoV-229E (1 TCID_50_/cell) for 30 h and treated with DTHIB (5 µM) or control diluent immediately after the adsorption period. Nuclei are stained with Hoechst (blue). Merge and zoom images are shown. Scale bar, 20 µm (zoom, 7 µm). **K** Levels of M-229E mRNA were analyzed by qRT-PCR in samples treated as in J. Error bars indicate means ± S.D. (n = 3). *p < 0.05; Student’s *t*-test. **L** MRC5-hACE2 cells infected with SARS-CoV-2 Omicron BA.1 variant (0.1 PFU/cell) were treated with different concentrations of DTHIB immediately after the adsorption period. Virus yield was determined at 22 h p.i. by plaque assay. Data, expressed as PFU/ml, represent the mean ± S.D. of samples from three independent experiments. ***p < 0.001; **p < 0.01; ANOVA test

Different inhibitors of the HSF1 pathway have been recently described [50]. An interesting new approach to selectively target nuclear HSF1 without disrupting the HSR cytoplasmic signaling, is represented by DTHIB (Direct-Targeted HSF1-InhiBitor, also named SISU-102) (Fig. 6A), which physically engages the HSF1-DBD and was shown to selectively stimulate HSF1 degradation in the nucleus of prostate cancer cells [51].

To ascertain whether DTHIB could downregulate HCoV-induced nuclear HSF1 in lung cells and determine its effect on HCoV replication, MRC-5 cells infected with HCoV-229E were treated with different concentrations of the drug after infection, and virus yield was determined at 24 h p.i.; in parallel, mock-infected MRC-5 cell viability was determined by MTT-assay. As shown in Fig. 6B, DTHIB was very effective in inhibiting HCoV-229E replication causing a > 3-log reduction in virus yield at non-cytotoxic concentrations. This effect was accompanied by a decrease in the level of HSF1-Ser326 phosphorylation and HCoV-nucleoprotein expression (Fig. 6C), as well as nuclear (but not cytoplasmic) levels of HSF1 (Fig. 6D, E), as previously reported [51]. Notably, DTHIB-treatment was effective in reducing viral yield and protein synthesis even when started as late as 10 h p.i., whereas pre-treatment of uninfected cells or treatment during the virus adsorption period had no effect on HCoV-induced HSF1-phosphorylation and virus replication (Fig. 6F, G), confirming that the drug is acting at late stages of virus replication. Similar results were obtained during HCoV-OC43 infection (Supplementary Fig. S6), demonstrating that DTHIB is effective in inhibiting both α- and β-HCoV replication.

To investigate the effect of DTHIB on the dynamic of HCoV-induced HSR activation, HSP levels were analyzed in MRC-5 cells at different times after virus infection. As shown in Fig. 6H, I, DTHIB inhibits virus-induced HSF1-dependent gene expression and viral replication up to 40 h p.i.. A remarkable reduction of viral RNA and protein levels was confirmed by qRT-PCR and immunofluorescence analysis at 24 h p.i. (Fig. 6J, K).

Finally, to determine whether DTHIB treatment was also effective against SARS-CoV-2 infection, MRC5-hACE2 cells infected with SARS-CoV-2 (Omicron BA.1, 0.1 PFU/cell) were treated with different concentrations of DTHIB after the 2 h adsorption period, and virus yield was determined at 22 h p.i. by plaque assay. As shown in Fig. 6L, DTHIB was very effective in inhibiting SARS-CoV-2 replication at low μM concentrations.

## Conclusions

Coronavirus infection is initiated by the binding of the spike glycoprotein to host receptors [18, 22]. After virus entry, the positive-sense RNA-genome hijacks the host ribosomes acting as a direct template for protein translation of two large ORFs (ORF1a, ORF1b) into the pp1a and pp1ab polyproteins, which are co-translationally and post-translationally processed by the ORF1a-encoded proteases into different non-structural proteins (nsps) that form the multi-subunit RNA replicase–transcriptase complex (RTC) containing several nsps, including the RNA-dependent RNA-polymerase, helicase and exonucleaseN proteins [18, 22]. The RTC-machinery needs to localize to modified intracellular membranes derived from the ER in the perinuclear region, where it drives the generation of negative-sense RNAs [(−)RNAs]. During replication, full-length (−)RNA copies of the genome are synthesized and used as templates for progeny RNA-genomes production; during transcription, a subset of 7–9 subgenomic RNAs, including those encoding structural proteins, is produced through discontinuous transcription, followed by viral mRNAs synthesis and translation. A complex and yet not well deciphered mechanism of assembly of both ribonucleocapsid structures and viral envelopes requires intense intracellular trafficking to and from the ER and the ERGIC, followed by release of the newly produced CoV-particles from the infected cell. Understanding the host-virus interactions enabling cells to sustain all the complex steps of the coronavirus lifecycle is pivotal to identifying potential targets for host-directed antiviral strategies.

We now show that both *Alpha*- and *Beta*- human coronaviruses, including seasonal HCoV-229E, HCoV-NL63 and HCoV-OC43, as well as highly-pathogenic SARS-CoV-2 Wuhan, Alpha, Delta and Omicron variants, are potent inducers of the proteostasis guardian HSF1, by selectively promoting HSF1-phosphorylation at a serine-residue (Ser326) crucial for transcriptional activity, and triggering a powerful and distinct HSF1-driven transcriptional response in infected cells at late stages of the viral lifecycle. It should be emphasized that the coronavirus-induced HSF1 transcriptional program appears to differ from the classical heat shock-induced HSF1-dependent gene expression profile in lung cells. As indicated above, in the classical heat-shock response HSF1 is generally considered to function as a guardian of cellular health; however, in addition to coronavirus infection, HSF1 was shown to drive diverse transcriptional programs in development, metabolism and cancer that are distinct from the classical HSR [36–38], indicating a more complex role of this fundamental transcription factor under different conditions. Beside the differences in the HSF1-dependent gene expression profile, a more interesting aspect is the distinct transcriptional/translational response observed in HCoV-induced, as compared to cellular stress-induced, activation of HSF1. In the context of HCoVs, which effectively shut-down the host translational machinery to prioritize the synthesis of viral proteins [22, 46], the accumulation of HSF1-dependent mRNAs does not consistently result in the synthesis of HSPs. Notably, our data revealed that the levels of classical molecular chaperone machines, including HSP60 and HSP90, which has been implicated in SARS-CoV-2 virion assembly [52], as well as ER-stress proteins such as GRP94 and GRP78, remained unaltered in cells infected with HCoVs despite substantial increase in mRNA levels; on the other hand, remarkably high levels of some HSF1-target gene products, including HSP70, HSPA6 and AIRAP, were found in HCoV-infected cells, revealing that selected host mRNAs are able to escape the virus-mediated translational block. On a more general ground, due to the complex and heterogeneous role of a variety of HSF1-dependent chaperones/co-chaperones during DNA and RNA viral infection [reviewed in 53], it is tempting to speculate that, under HSF1-inducing stress conditions such as hyperthermia, viruses that adopt diverse strategies to control the host translational machinery may allow differential translation of selected HSPs.

Interestingly, in the case of HCoVs, silencing experiments demonstrate that HSR activation does not merely reflect a cellular response to the virus-induced proteotoxic stress caused by the abundant synthesis and intracellular trafficking of foreign proteins, but that HCoV activate and hijack the HSF1-pathway for their own gain. Notably, post-infection treatment with the recently described HSF1-inhibitor DTHIB/SISU-102, which selectively targets nuclear HSF1, was found to be highly effective in inhibiting sHCoV and SARS-CoV-2 omicron BA.1 replication, remarkably reducing the production of HCoV progeny particles in lung cells.

Whereas the role of different canonical and non-canonical HSPs in HCoV replication remains to be elucidated, it could be argued that, due to the complexity of coronavirus replication strategies, a number of different chaperones/cochaperones may be required for an efficient control of the host proteostasis by the invading pathogen. On the other hand, considering the high levels of HSPs with distinct prosurvival/anti-apoptotic roles, including HSPA6, AIRAP and HSP70 [43, 54, 55], detected in HCoV-infected cells, it could be hypothesized that coronaviruses, generally characterized by a slow lifecycle requiring endomembrane compartments functional and structural alterations [56], may stimulate an HSF1-driven prosurvival pathway in order to avoid that early death of the host-cell could hamper efficient progeny production. It is conceivable that both prosurvival HSPs and different types of molecular chaperones are needed throughout the HCoV lifecycle. Future studies aiming at the systematic depletion of different HSF1-target genes will be crucial for identifying the specific molecular chaperones and/or pro-survival HSPs that are co-opted by human coronaviruses within the host cell during infection. This approach will not only enhance our understanding of the virus-host interaction dynamics, but could also potentially reveal novel therapeutic targets for disrupting viral exploitation of host cellular functions.

Altogether the results identify HSF1 as a major player in coronavirus replication, opening new scenarios for the search of innovative antiviral strategies in the treatment of coronavirus infections.

## Materials and methods

### Cell culture and treatments

Human normal lung MRC-5 fibroblasts (American Type Culture Collection, ATCC, CCL-171), African green monkey kidney Vero cells (ATCC, CCL-81), Vero E6 cells (ATCC, CRL-1586), HeLa cells and Caco-2 cells (ATCC) were grown at 37 °C in a 5% CO_2_ atmosphere in EMEM (MRC-5 cells) or DMEM (Vero, Vero E6, Caco-2 and HeLa cells) medium, supplemented with 10% fetal calf serum (FCS), 2 mM glutamine and antibiotics. Generation of Caco-2 hACE2 cells stably expressing the hACE2 receptor [57], HeLa cells stably transfected with pSUPER-HSF1i/pcDNA3 (HeLa-HSF1i) or control (HeLa wild-type) plasmids [49], and Vero-hACE2-TMPRSS2 stably expressing the hACE2 and hTMPRSS2 receptors [58] was described previously. For heating procedures, cells were subjected to heat shock at the indicated temperature in a precision water bath W14 (Grant Instruments) [15]. DTHIB (Direct Targeted HSF1 Inhibitor) (Selleckchem) dissolved in DMSO stock solution, was diluted in culture medium, added to infected cells after the virus adsorption period, and maintained in the medium for the duration of the experiment. Bortezomib (BTZ, Selleckchem) was dissolved in DMSO and diluted in culture medium immediately before use. Controls received equal amounts of DMSO vehicle, which did not affect cell viability or virus replication. Cell viability was determined by the 3-(4,5-dimethylthiazol-2-yl)-2,5-diphenyltetrazolium bromide (MTT) to MTT formazan conversion assay (Sigma-Aldrich), as described [59]. The 50% lethal dose (LD_50_) was calculated using Prism 8.0 software (Graph-Pad Software Inc.). Microscopical examination of mock-infected or virus-infected cells was performed daily to detect virus-induced cytopathic effect and possible morphological changes and/or cytoprotection induced by the drug. Microscopy studies were performed using a Leica DM-IL microscope and images were captured on a Leica DC 300 camera using Leica Image-Manager500 software.

### Generation of the MRC5-hACE2 stable cell line

Lentiviral particles to generate hACE2 stably expressing cells were produced by co-transfection of 293 T cells with the VSV-G encoding plasmid [60], the pCMVR 8.74 lentiviral packaging plasmid (a kind gift from R. Piva, University of Turin, Italy) and the pLENTI hACE2 PURO vector (a gift from Raffaele De Francesco, INGM, Milan; Addgene plasmid #155295). At 48 h after transfection, supernatants were collected, filtered through 0.45-mm membranes and used to infect MRC-5 cells. At 72 h after infection, selection with puromycin (0.75 μg/ml) was started, and after 5 days in selective medium resistant pools of MRC-5 cells were obtained.

### Virus infection and titration

Human seasonal coronaviruses HCoV-229E (ATCC), HCoV-OC43 (ATCC) and HCoV-NL63 (strain Amsterdam-1, a kind gift from Lia van der Hoek, University of Amsterdam), were used for this study [61]. For virus infection, confluent MRC-5 (229E and OC43) or Caco-2 hACE2 (NL63) cell monolayers were infected with HCoV for 1 (229E and OC43) or 2 (NL63) hours at 33 °C at a multiplicity of infection (m.o.i.) of 0.1 or 1 TCID_50_ (50% tissue culture infectious dose)/cell. After the adsorption period, the viral inoculum was removed, and cell monolayers were washed three times with phosphate-buffered saline (PBS). Cells were maintained at 33 °C in growth medium containing 2% FCS. Virus yield was determined at different times after infection by TCID_50_ infectivity assay, as described previously [62]. The 50% inhibitory concentration (IC_50_) of the compound tested was calculated using Prism 8.0 software.

SARS-CoV-2 viruses used in this study were: the original Wuhan strain (B29) from Lance Turtle (University of Liverpool) and David Matthews and Andrew Davidson (University of Bristol); variant alpha (B.1.1.7) from Ian Goodfellow (University of Cambridge) and variants delta (B.1.617.2) and omicron (BA.1) from Ravindra Gupta (University of Cambridge). Virus stocks were prepared in Vero hACE2-TMPRSS2 cells. Cells were infected with SARS-CoV-2 at a m.o.i. of 0.01 and incubated for 3 days. Virus stock was harvested by three freeze–thaw cycles followed by 5 min 300×*g* spin to remove cell debris. Virus yield was determined by plaque assay.

For MRC5-hACE2 cells infection, cells were infected with the different SARS-CoV-2 variants [0.1 PFU (plaque forming units)/cell] for 40 h and levels of HSF1 phosphorylation were analyzed. For DTHIB experiments, MRC5-hACE2 cells were seeded into 96-well plates and, after 24 h, were infected with the Omicron BA.1 variant at a m.o.i. of 0.1 PFU/cell for 2 h at 37 °C. After this time, media was changed into inhibitor/DMSO containing media and cells were incubated for further 20 h at 37 °C. After incubation cells and supernatants were freeze-thawed three times to release all intracellular viruses and then titrated by plaque assay into Vero hACE2-TMPRSS2 cells. After 1 h incubation wells were layered with media containing 2% FBS and 0.05% agarose and then incubated for 3 days. Cells were fixed with 4% formaldehyde, stained with 0.1% toluidine blue and plaques were counted using a phase contrast microscope.

Rhabdovirus VSV (vesicular stomatitis virus) Indiana serotype (Orsay) was kindly provided by Dr. E. Rodriguez-Boulan (Cornell University Medical College, New York). For virus infection, confluent HeLa cell monolayers were infected with VSV for 1 h at 37 °C at different m.o.i.. After the adsorption period, the viral inoculum was removed, and cell monolayers were washed three times with PBS. Cells were maintained at 37 °C in growth medium containing 2% FCS. Virus yield was determined by TCID_50_ infectivity assay [63].

### Protein analysis, puromycinylation and Western blot

For analysis of proteins, whole-cell extracts (WCE) were prepared after lysis in High Salt Buffer (HSB) [57]. Nuclear and cytoplasmic extracts were prepared as described [64]. For SARS-CoV-2 experiments, cells were lysed with 1 × Laemmli buffer (10% glycerol, 2% SDS, 63 mM TrisHCl pH 6.8, bromophenol blue and 50 mM DTT) and then treated with Benzonase Nuclease (70664, Millipore) for 30 min before boiling and loading onto gels. For Western blot analysis, cell extracts were separated by SDS-PAGE under reducing or non-reducing conditions and blotted to a nitrocellulose membrane. Membranes were incubated with the selected antibodies, followed by incubation with peroxidase-labeled anti-rabbit, anti-mouse or anti-goat IgG. Primary and secondary antibodies used are listed in Supplementary Table S3. To label nascent polypeptides, MRC-5 cells were treated with puromycin (2.5 µg/ml) 30 min prior to harvesting. WCE were prepared as described [57]. After immunoblotting, membranes were stained with Ponceau S solution to assess the steady-state proteomes and then hybridized with anti-puromycin antibodies to detect puromycinylated polypeptides. Quantitative evaluation of proteins was determined as described [65].

### Electrophoretic mobility shift assay (EMSA)

The 35-bp HSP70-HSE DNA probe was described previously [66]. WCE (10 μg) prepared after lysis in high-salt extraction buffer were incubated with a ^32^P-labeled HSE DNA probe [67] followed by analysis of DNA-binding activity by EMSA. Binding reactions were performed as described [64]. Complexes were analyzed by nondenaturing 4% polyacrylamide gel electrophoresis. To determine the specificity of HSF-DNA complexes, WCE were preincubated with different dilutions of anti-HSF1 (sc-9144, Santa Cruz Biotechnology) or anti-HSF2 (sc-13056, Santa Cruz Biotechnology) antibodies for 15 min before electromobility supershift assay [68].

### Immunofluorescence microscopy

MRC-5 cells infected with HCoV-OC43 or HCoV-229E were grown in 8-well chamber slides (Lab-Tek II) and, after the adsorption period, were treated with DTHIB, or vehicle for 30 h. Cells were fixed, permeabilized and processed for immunofluorescence as described [57] using selected antibodies, followed by decoration with Alexa Fluor 555- or 488-conjugated antibodies (Molecular Probes, Invitrogen). Nuclei were stained with Hoechst 33342 (Molecular Probes, Invitrogen). For confocal microscopy, images were acquired on Olympus FluoView FV-1000 confocal laser scanning system (Olympus America Inc., Center Valley, PA) and analyzed using Imaris (v6.2) software (Bitplane, Zurich, Switzerland). Images shown in all figures are representative of at least three random fields (scale-bars are indicated).

### RNA extraction, gene expression and real-time PCR analysis

Total RNA from mock-infected or infected cells was prepared using ReliaPrep RNA Cell Miniprep System (Promega) and reverse transcription was performed with PrimeScript RT Reagent Kit (Takara) according to the manufacturer’s protocol. Gene expression of 84 heat shock genes in cells mock-infected or infected with HCoV-229E, or exposed to heat stress was analyzed using Human Heat Shock Proteins and Chaperones RT^2^ Profiler PCR array (Qiagen). Real-time PCR analyses were performed with specific primers, listed in Supplementary Table S4, using SsoAdvanced Universal SYBR Green Supermix (CFX96, Bio-Rad). Relative quantities of selected mRNAs were normalized to L34 [61]. All reactions were made in triplicate using samples derived from at least three biological repeats.

### siRNA interference

Two siRNA duplex target sequences (siHSF1_1_ and siHSF1_2_) and their scrambled control (scrRNA) (QIAGEN) were used for HSF1-silencing (Supplementary Table S2). Transfections were performed using jetPRIME Transfection Reagent, according to the manufacturer’s instructions. In brief, cells were plated on 35-mm wells (2.5 × 10^5^ cells/well) and, after 24 h, were transfected with 50 nM of the indicated siRNAs (siHSF1_1_ and siHSF1_2_) or scrRNA. After 24 h, cells were washed twice with culture medium and transfection was repeated as above. At 24 h after the second transfection, siRNAs were removed, and cells were washed twice with culture medium before HCoV-229E infection.

### HCoV genomic RNA transfection

For HCoV genomic RNA transfection experiments, MRC-5 cell monolayers were infected with HCoV-229E or HCoV-OC43 for 1 h at 33 °C at an m.o.i. of 0.1 TCID_50_/cell and HCoV genomic RNA was extracted from the supernatants at 24 h p.i. using TRIzol-LS reagent (Life Technologies) as described in the manufacturer protocol. HeLa wild-type or HeLa-HSF1i cell monolayers were mock-transfected or co-transfected with HCoV genomic RNA (1 μg/ml) and pCMV-GFP vector (Clontech) using TransIT-mRNA Transfection Kit (Mirus Bio) at 33 °C [61]. After 4 h, transfection medium was removed and cells were maintained at 33 °C in growth medium containing 2% FCS. After 48 h (229E) or 72 h (OC43), culture supernatants were collected for virus progeny titer determination, and cell monolayers were processed by Western blot analysis.

### Statistical analysis

Statistical analyses were performed using Prism 8.0 software (GraphPad Software). Comparisons between two groups were made using Student's *t* test; comparisons among groups were performed by one-way ANOVA with Bonferroni adjustments. *p* values ≤ 0.05 were considered significant. Data are expressed as the means ± standard deviations (S.D.); the results shown are representative of at least two independent experiments, each in duplicate or triplicate.

## Supplementary Information

Below is the link to the electronic supplementary material.

Supplementary file1 (PDF 8311 KB)

## Data Availability

Data will be made available on reasonable request.
