# Supplementary material for: Human coronaviruses activate and hijack the host transcription factor HSF1 to enhance viral replication
Source: Cell Mol Life Sci. 2024 Sep 7;81(1):386. doi: 10.1007/s00018-024-05370-5 (PMC11380654; doi:10.1007/s00018-024-05370-5)
Supplement: Supplementary file 1 — Supplementary file1 (PDF 8311 KB) [file 18_2024_5370_MOESM1_ESM.pdf]

Supplementary Information

Human coronaviruses activate and hijack the host transcription factor HSF1 to enhance viral replication

Silvia Pauciullo[1], Anna Riccio[1], Silvia Santopolo[1], Anna Albecka[2], Guido Papa[2], Leo C. James[2], Sara Piacentini[1], Giulia Lanzilli[3], Antonio Rossi[3] and M. Gabriella Santoro[1,3]

[1]Department of Biology, University of Rome Tor Vergata, Rome, Italy; [2]MRC Laboratory of Molecular Biology, Francis Crick Avenue, Cambridge CB2 0QH, UK; [3]Institute of Translational Pharmacology, CNR, Rome, Italy.

Supplementary Table 1. Expression profile of 84 HSF1-target genes in HCoV-229E infected cells

GENE	Fold Change	*p*-value	GENE	Fold Change	*p*-value
ATF6	2,24	0,004042	DNAJC21	1,79	0,000057
BAG1	2,86	0,002787	DNAJC3	13,52	0,000123
BAG2	2,95	0,000159	DNAJC4	1,09	0,072608
BAG3	8,75	0,000143	DNAJC5	2,74	0,000118
BAG4	2,26	0,003037	DNAJC5B	1,94	0,433188
BAG5	3,41	0,0003	DNAJC5G	2,59	0,058501
CCS	1,41	0,007209	DNAJC6	1,24	0,550764
CCT2	2,43	0,006482	DNAJC7	2,49	0,004597
CCT3	3	0,006755	DNAJC8	1,37	0,023288
CCT4	3,51	0,000201	DNAJC9	1,03	0,802655
CCT5	2,34	0,000076	HSF1	1,68	0,009516
CCT6A	1,79	0,000517	HSF2	4,05	0,000862
CCT6B	1,97	0,00996	HSF4	1,39	0,145565
CCT7	1,52	0,003472	HSP90AA1	6,53	0,00124
COQ8A	1,95	0,018829	HSP90AB1	3,57	0,000081
CRYAA	0,93	0,972409	HSP90B1	5,5	0,000083
CRYAB	7,64	0,000045	HSPA14	2,58	0,000824
DNAJA1	3,42	0,000447	HSPA1A	26,45	0,005915
DNAJA2	2,22	0,001657	HSPA1B	54,66	0,000652
DNAJA3	2,95	0,00021	HSPA1L	1,49	0,423684
DNAJA4	37,68	0,001242	HSPA2	0,8	0,08965
DNAJB1	21,92	0,003122	HSPA4	2,61	0,001488
DNAJB11	5,54	0,000045	HSPA4L	10,03	0,000241
DNAJB12	2,43	0,000333	HSPA5	12,74	0,000184
DNAJB13	1,21	0,266541	HSPA6	1674,96	0,002617
DNAJB14	3,36	0,000008	HSPA8	2,05	0,001686
DNAJB2	2,87	0,000061	HSPA9	6,1	0,000005
DNAJB5	3,41	0,000003	HSPB1	1,62	0,181349
DNAJB6	2,57	0,000329	HSPB2	0,67	0,64913
DNAJB7	1,35	0,13324	HSPB3	0,88	0,584931
DNAJB8	2,14	0,227409	HSPB6	0,39	0,013922
DNAJB9	15,16	0,00002	HSPB7	0,64	0,160068
DNAJC1	2,88	0,000067	HSPB8	5,99	0,000064
DNAJC10	3,28	0,000933	HSPD1	4,46	0,000065
DNAJC11	2,11	0,00018	HSPE1	1,78	0,001644
DNAJC12	12,54	0,000116	HSPH1	13,63	0,000432
DNAJC13	1,67	0,000871	PFDN1	0,9	0,433629
DNAJC14	2,2	0,000264	PFDN2	1,36	0,002902
DNAJC15	1,14	0,014643	SERPINH1	3,74	0,00152
DNAJC16	3,69	0,000304	SIL1	2,49	0,001381
DNAJC17	1,25	0,48577	TCP1	3,14	0,000443
DNAJC18	1,97	0,006605	TOR1A	1,17	0,068212

MRC-5 cells infected with HCoV-229E (0.1 $TCID_{50}$/cell) for 24h. For details see legend Fig. 3A.

Supplementary Table 2. Expression profile of 84 HSF1-target genes in heat-shocked cells

GENE	Fold Change	*p*-value	GENE	Fold Change	*p*-value
ATF6	1,47	0,036919	DNAJC21	0,96	0,629384
BAG1	2,36	0,000475	DNAJC3	1,91	0,000467
BAG2	0,82	0,279689	DNAJC4	1,48	0,002066
BAG3	7,34	0,000124	DNAJC5	1,01	0,931602
BAG4	0,85	0,315173	DNAJC5B	1,39	0,176319
BAG5	1,33	0,115042	DNAJC5G	12,38	0,063994
CCS	2,01	0,00668	DNAJC6	2,29	0,01689
CCT2	1,13	0,372228	DNAJC7	0,97	0,738533
CCT3	1,34	0,221978	DNAJC8	1,05	0,75302
CCT4	1,53	0,078246	DNAJC9	1,4	0,069386
CCT5	0,74	0,009083	HSF1	1,14	0,171801
CCT6A	1,61	0,005258	HSF2	1,68	0,004032
CCT6B	1,75	0,067021	HSF4	5,05	0,006061
CCT7	1,08	0,890066	HSP90AA1	3,48	0,000019
COQ8A	3,15	0,002652	HSP90AB1	1,26	0,005016
CRYAA	10,5	0,06443	HSP90B1	1,9	0,001505
CRYAB	7,65	0,000006	HSPA14	0,67	0,027158
DNAJA1	5,52	0,000065	HSPA1A	95,02	0,000005
DNAJA2	1,51	0,123754	HSPA1B	155,67	0,000006
DNAJA3	0,96	0,734253	HSPA1L	8,16	0,010231
DNAJA4	13,25	0,000001	HSPA2	0	0,05966
DNAJB1	40,42	0,000005	HSPA4	1,48	0,002033
DNAJB11	2,18	0,008582	HSPA4L	23,25	0,000588
DNAJB12	1,23	0,215473	HSPA5	3,02	0,000101
DNAJB13	7,36	0,025194	HSPA6	312,55	0,000002
DNAJB14	1,15	0,305747	HSPA8	1,82	0,002618
DNAJB2	2,18	0,000273	HSPA9	0,65	0,007138
DNAJB5	0,65	0,135038	HSPB1	3,14	0,00004
DNAJB6	1,65	0,009224	HSPB2	1,56	0,154934
DNAJB7	2,5	0,087585	HSPB3	0,41	0,020257
DNAJB8	0,92	0,489083	HSPB6	1,04	0,60288
DNAJB9	3,51	0,000137	HSPB7	0,37	0,000011
DNAJC1	1,14	0,30654	HSPB8	1,36	0,219214
DNAJC10	1,19	0,459702	HSPD1	2,13	0,000165
DNAJC11	0,7	0,068169	HSPE1	2,36	0,000012
DNAJC12	4,17	0,000144	HSPH1	5,11	0,000044
DNAJC13	1,05	0,9834	PFDN1	1,13	0,356393
DNAJC14	1,39	0,00831	PFDN2	0,74	0,005894
DNAJC15	1,24	0,071779	SERPINH1	1,83	0,000028
DNAJC16	1,12	0,672599	SIL1	1,21	0,039457
DNAJC17	1,38	0,008337	TCP1	1,31	0,019544
DNAJC18	0,97	0,741256	TOR1A	0,79	0,166842

MRC-5 cells exposed to heat-shock (43°C, 40 min, 1.5 h recovery). For details see Fig. S2 legend.

Supplementary Table 3. Antibodies used

Antibody	Source	Catalogue Number
HCoV-229E Spike (P)	LGC NAC Company	PAB21477-100
HCoV-229E Nucleocapsid (P)	Sino Biological	40640-T62
HCoV-OC43 Nucleocapsid (P)	Sino Biological	40643-T62
KDEL (M)	Enzo Life Sciences	ADI-SPA-827
HCoV-NL63 Nucleocapsid (P)	Sino Biological	40641-T62
VSV-G protein (M)	Sigma-Aldrich	V5507
SARS-CoV-2 spike (M)	LGC NAC Company	MAB12444
Puromycin (M)	Sigma-Aldrich	MABE343
dsRNA (M)	SCICONS	10010200
α-Tubulin (M)	Sigma-Aldrich	T5168
HSF1 (P)	Enzo Life Sciences	ADI-SPA-901
HSF1 (phospho-S121) (P)	Tebubio	A8041
HSF1 (phospho-S303) (P)	abcam	ab47369
HSF1 (phospho-S326) (P)	abcam	ab76076
β-Actin (P)	Sigma-Aldrich	A2066
ZFAND2A (P)	Sigma-Aldrich	HPA019469
GAPDH (P)	Cusabio	CSB-PA00025A0Rb
HSP70 (M)	Enzo Life Sciences	ADI-SPA-810
Histone H3 (P)	abcam	ab1791
HSP90β (P)	Santa Cruz Biotechnology	sc-1057
HSP90 (P)	Cell Signaling	4877
HSP60 (P)	StressMarq	SPC-105
GFP (M)	CUSABIO	CSB-MA000051M0m
ACE2 (M)	Santa Cruz Biotechnology	sc-73668
Alexa Fluor 488 goat anti-mouse	Invitrogen	A11001
Alexa Fluor 555 goat anti-rabbit	Invitrogen	A21428
Goat Anti-Mouse IgG (H+L), HRP	Jackson ImmunoResearch	115-035-003
Goat Anti-Rabbit IgG (H+L), HRP	Jackson ImmunoResearch	111-035-003
Mouse Anti-Goat IgG (H+L), HRP	Santa Cruz Biotechnology	sc-2354

(**M**): Monoclonal; (**P**): Polyclonal

Supplementary Table 4. Primers and siRNAs used

Primers used for mRNA gene expression		
**Gene**	**Primer direction**	**Primer sequence**
AIRAP	Forward	5′-TCATTTTCCATACGCTGCAC-3′
	Reverse	5′-CTGTGGTCCAAAGGGTGTCT-3′
COX-2	Forward	5′-TTGCTGGCAGGGTTGCTGGTGGTA-3′
	Reverse	5′-CATCTGCCTGCTCTGGTCAATGGAA-3′
HSP701A	Forward	5′-CTACAAGGGGGAGACCAAGG-3′
	Reverse	5′-TTCACCAGCCTGTTGTCAAA-3′
NKRF	Forward	5′-CCAAACCTTCCAAAGGTCAA-3′
	Reverse	5′-CAGGGTTCCCACTGTCAAAA-3′
HCoV-229E	Forward	5′- TTCCGACGTGCTCGAACTTT-3′
	Reverse	5′- CCAACACGGTTGTGACAGTGA-3′
L34	Forward	5′-GGCCCTGCTGACATGTTTCTT-3′
	Reverse	5′-GTCCCGAACCCCTGGTAATAGA-3′

siRNAs sequences	
**siRNA**	**Target sequence**
**$siHSF1_1$**	5′-TACCCAAGTACTTCAAGCACA-3′
**$siHSF1_2$**	5′-CAGTGACCACTTGGATGCTAT-3′

SUPPLEMENTARY FIGURE LEGENDS

Figure S1. HCoV-induced HSF1 phosphorylation and expression of HSF1-target genes is time- and m.o.i.-dependent, and is not mimicked by VSV infection. (**A**) Schematic representation of the experimental protocol (top panel). Whole-cell extracts (WCE) from samples mock-infected (Mock) or infected with HCoV-229E (0.1 $TCID_{50}$/cell) were analyzed for pHSF1 (Ser326), viral nucleocapsid (N)and α-tubulin protein levels at different times post infection (p.i.) by IB (bottom panels). (**B**) Immunoblot analysis of pHSF1 (Ser326), VSV-G glycoprotein and β-actin levels in HeLa cells mock-infected (0) or infected with VSV at different m.o.i. (0.1, 0.5 or 1 PFU/cell) or exposed to heat-stress (HS, 43°C, 40 min) (top panels). pHSF1-Ser326 protein levels were determined by densitometric analysis using ImageJ software (bottom panel). (**C**) MRC-5 cells were mock-infected or infected with HCoV-229E (1 $TCID_{50}$/cell) and levels of viral membrane (M-229E), HSP70 and AIRAP mRNA were analyzed at different times p.i. by qRT-PCR. (**D**) Total mRNA was extracted from MRC-5 cells mock-infected or infected with HCoV-229E (0.1 or 1 $TCID_{50}$/cell) for 24h and analyzed for M-229E, HSP70 and AIRAP gene expression by qRT-PCR. The fold increase was calculated by comparing the induction of M-229E, HSP70 and AIRAP in each sample to the relative control at 0h p.i. (C) or to the mock-infected control (D), which were arbitrarily set to 1. Error bars indicate means ± S.D. (n=3). * = $p < 0.05$; Student's *t*-test (B, C); ANOVA test (D).

Figure S2. Heat-shock and HCoV-229E infection turn on distinct HSF1-driven transcriptional programs in human lung cells. (**A-C**) Expression profile of selected HSF1-target genes affected by heat-shock (HS: 43°C, 40 min, 1.5h recovery) in MRC-5 cells as determined by qRT-PCR array (PAHS-076ZD-2-Qiagen). Heat Map (A) and Volcano plot (B) of 84 human HSPs and chaperones/cochaperones gene expression. In (A) each row represents a single gene, each column represents the mean of triplicate samples. The gradual color ranging from blue to red represents the mRNA expression level (Z-score). In the Volcano plot (B) fold regulation threshold is set to 2 and *p*- value cut off is 0.05; each dot represents a gene: red and blue dots indicate genes respectively up- and down-regulated more than 2-fold. Selected HSPs and chaperones/cochaperones genes whose expression is highly induced by HS are shown in (C). (**D,E**) Venn diagram (D) and comparison (E) of significantly ($p<0.05$) deregulated genes in MRC-5 cells heat-stressed as in (A) or infected with HCoV-229E (0.1 $TCID_{50}$/cell) for 24h.

Figure S3. **HCoV infection selectively induces canonical and non-canonical HSP expression in human lung cells.** (**A**) Levels of HSP90, GRP94, GRP78, HSP70, HSPA6, HSP60, AIRAP and viral spike protein shown in Fig. 3F were determined by densitometric analysis using ImageJ software, normalized to β-actin and expressed as arbitrary units (A.U.). (**B**) Levels of GRP94, GRP78, HSP70 and viral nucleocapsid (N-229E) protein shown in Fig. 3H were determined by densitometric analysis as in A. Error bars indicate means ± S.D. (n=3). * = $p < 0.05$; ** = $p < 0.01$; Student's *t*-test.

Figure S4. HCoV-OC43 and HCoV-NL63 infection induces the expression of HSF1-target genes in human cells. (**A, C**) Schematic representation of the experimental protocol (top panels). Immunoblot analysis of HSPA6, HSP60, AIRAP, viral nucleocapsid (N), α-tubulin and β-actin protein levels in MRC-5 (A) and Caco-2 hACE2 (C) cells mock-infected (-) or infected (+) with HCoV-OC43 (A) for 24h or HCoV-NL63 (C) for 72h at a m.o.i. of 0.1 $TCID_{50}$/cell (bottom panels). (**B, D**) Relative amounts of HSPA6, HSP60 and AIRAP proteins, normalized to α-tubulin (B) or to β-actin (D), were determined by densitometric analysis using ImageJ software. Error bars indicate means ± S.D. (n=3). * = $p < 0.05$; Student's *t*-test.

Figure S5. Effect of different SARS-CoV-2 variants on HSF1 phosphorylation in MRC5-hACE2 cells. (**A**) Schematic representation of the experimental protocol. (**B, C**) MRC5-hACE2 cells were mock-infected or infected with the Wuhan, Alpha and Delta SARS-CoV-2 variants (0.1 PFU/cell) for 40h. Equal amounts of whole-cell extracts (10 μl) were analyzed for levels of HSF1-Ser326, HSF1 and β-actin by IB (B). Levels of HSF1-Ser326 phosphorylation were determined after normalizing to β-actin and expressed as fold induction of the mock-infected control, which was arbitrarily set to 1 (C).

Figure S6. DTHIB treatment inhibits HCoV-OC43 replication. (**A**) MRC-5 cells mock-infected or infected with HCoV-OC43 (0.1 $TCID_{50}$/cell) were treated with different concentrations of DTHIB immediately after the adsorption period. (**B, C**) MRC-5 cells were treated with 10 μM DTHIB (filled bars) or control vehicle (C, empty bars) at 3h before infection (PRE), only during the adsorption period (ADS), immediately after the adsorption period (POST 0h) or at 10h after infection (POST 10h) with HCoV-OC43 at a m.o.i. of 0.1 $TCID_{50}$/cell (B) or 1 $TCID_{50}$/cell (C). (**A-C**) Virus yield was determined at 24h p.i. by $TCID_{50}$ infectivity assay. Data, expressed as $TCID_{50}$/ml, represent the mean ± S.D. (n=3). * = $p < 0.05$, ** = p 0.01, *** = $p < 0.001$; ANOVA test.

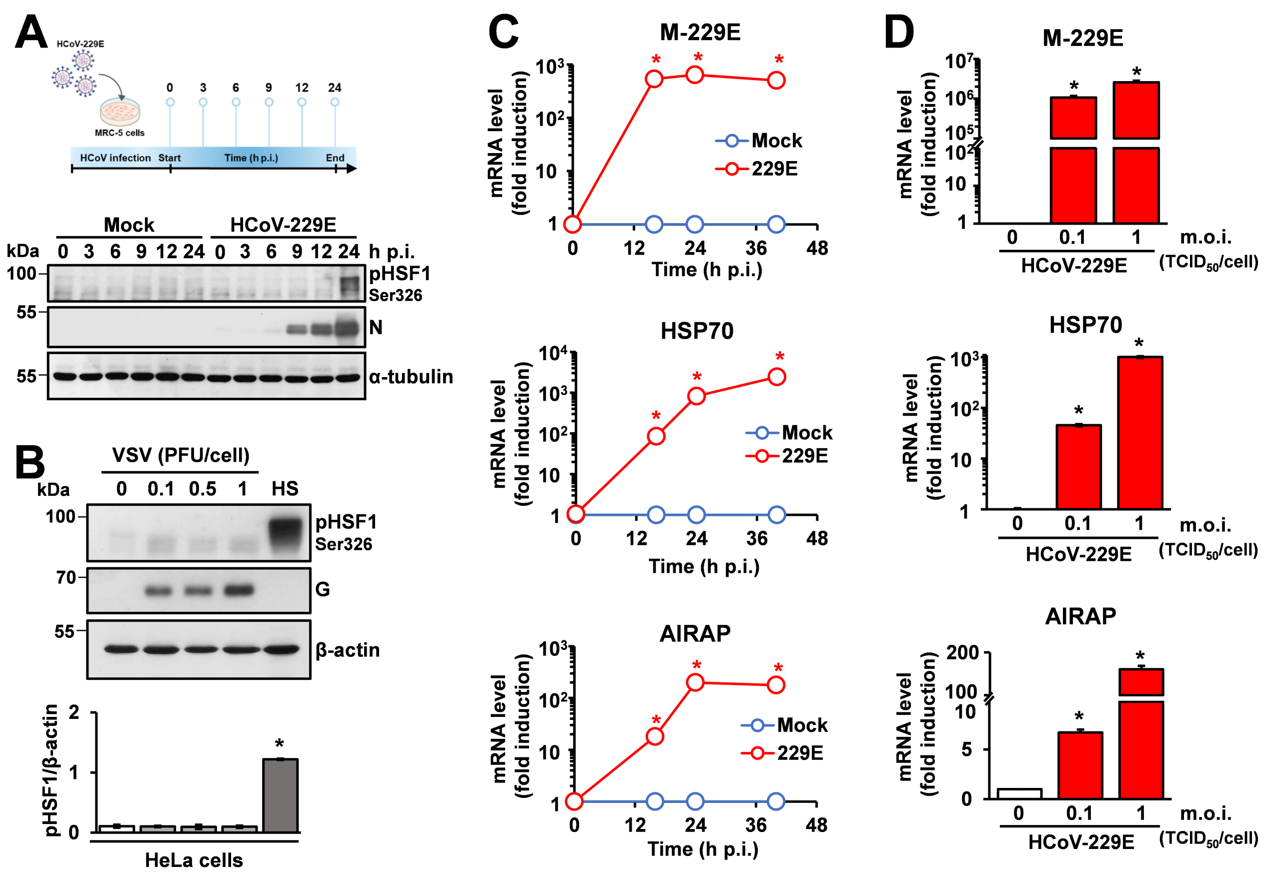

A
HCoV-229E
MRC-5 cells
0 3 6 9 12 24
HCoV infection Start Time (h p.i.) End
Mock
HCoV-229E
kDa
0 3 6 9 12 24 0 3 6 9 12 24 h p.i.
100
pHSF1 Ser326
55
N
55
α-tubulin
B
VSV (PFU/cell)
kDa
0 0.1 0.5 1 HS
100
pHSF1 Ser326
70
G
55
β-actin
pHSF1/β-actin
HeLa cells
C
M-229E
mRNA level (fold induction)
Mock
229E
Time (h p.i.)
HSP70
AIRAP
D
M-229E
HSP70
AIRAP
m.o.i.
HCoV-229E
(TCID50/cell)

Figure S1

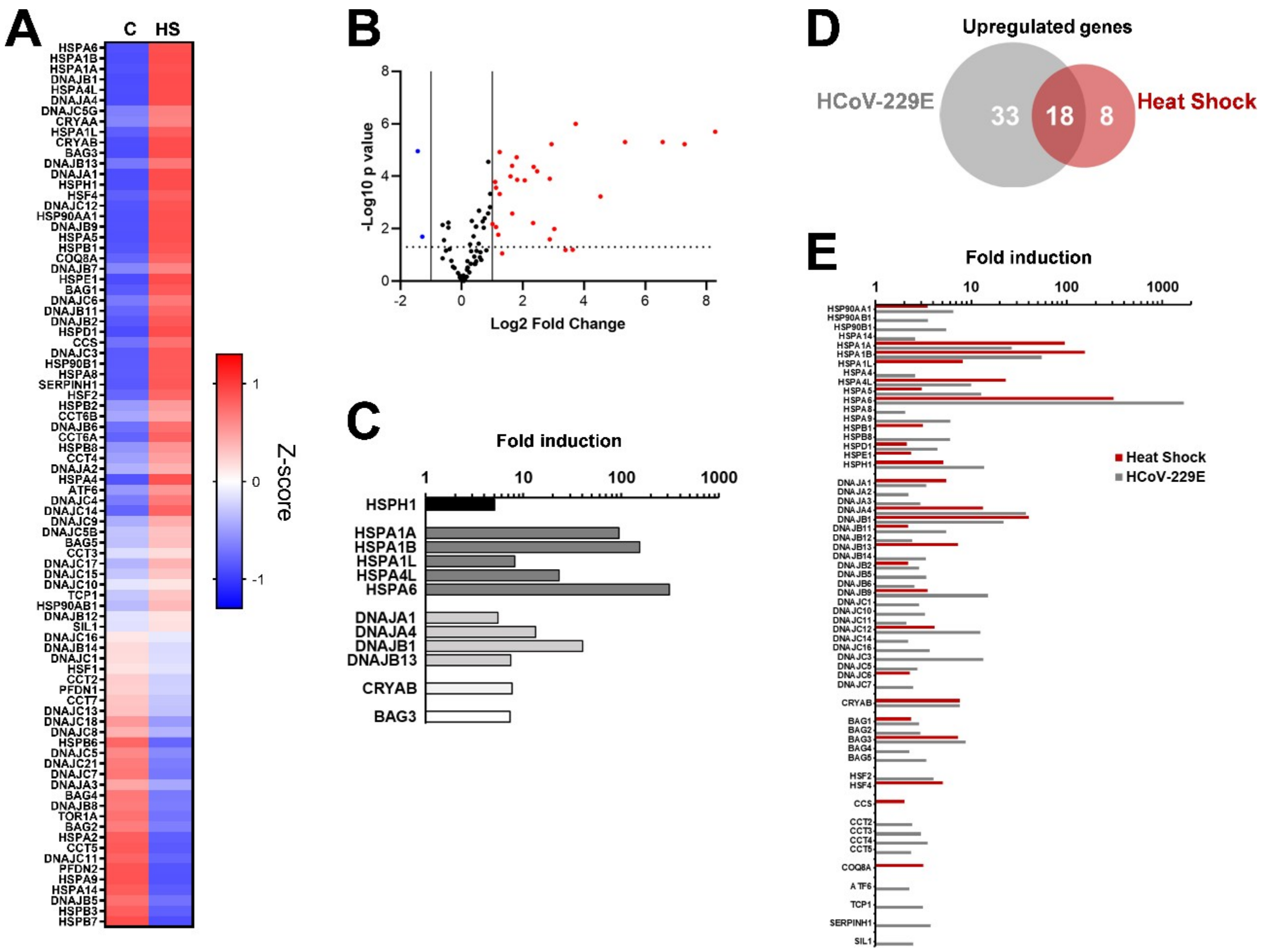
A
C
HS
Z-score
B
-Log10 p value
Log2 Fold Change
C
Fold induction
HSPH1
HSPA1A
HSPA1B
HSPA1L
HSPA4L
HSPA6
DNAJA1
DNAJA4
DNAJB1
DNAJB13
CRYAB
BAG3
D
Upregulated genes
HCoV-229E
Heat Shock
33
18
8
E
Fold induction
Heat Shock
HCoV-229E

Figure S2

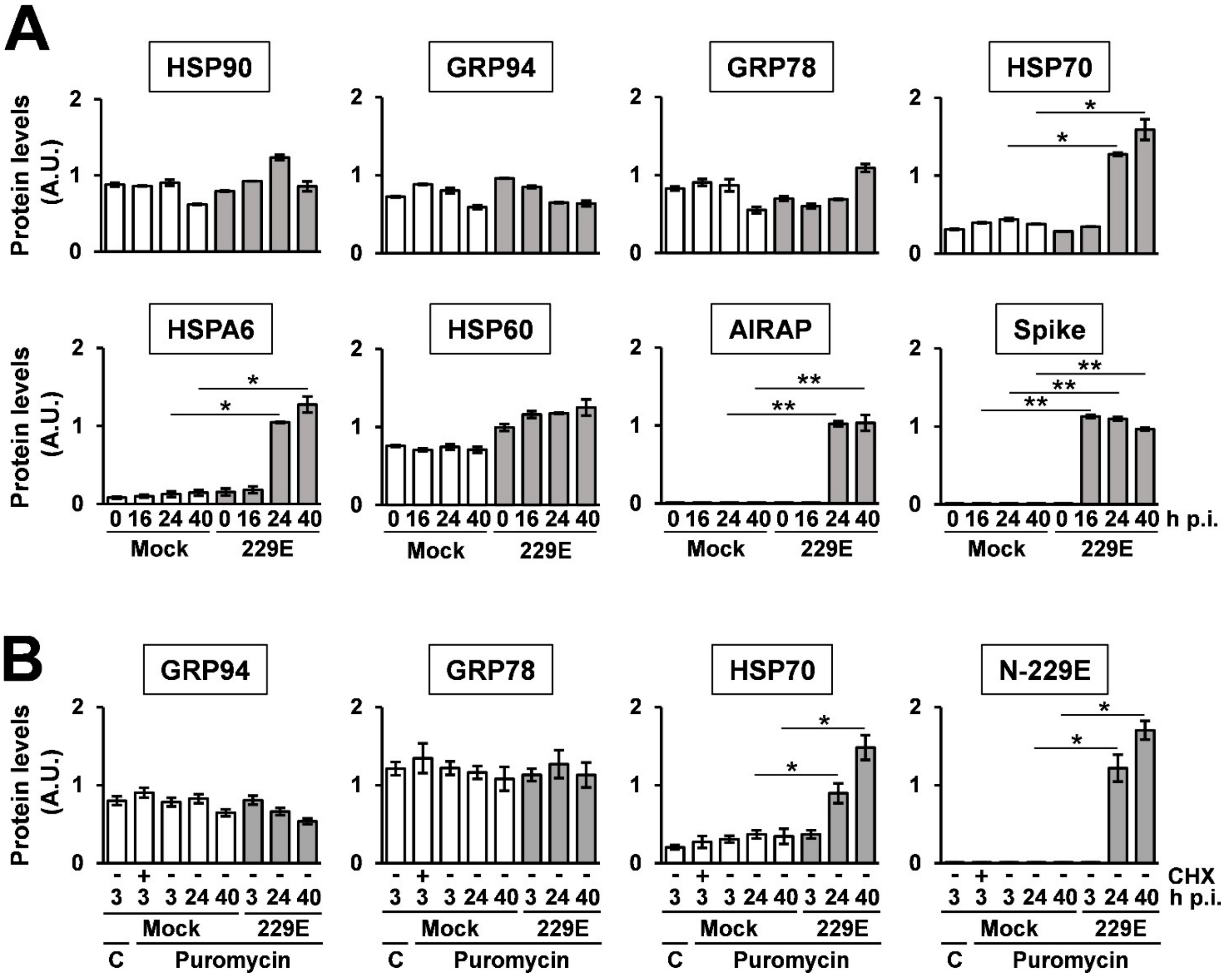
A
B
HSP90
GRP94
GRP78
HSP70
HSPA6
HSP60
AIRAP
Spike
N-229E
Protein levels (A.U.)
0 16 24 40 0 16 24 40 h p.i.
Mock
229E
CHX
3 3 3 24 40 3 24 40 h p.i.
C
Puromycin

Figure S3

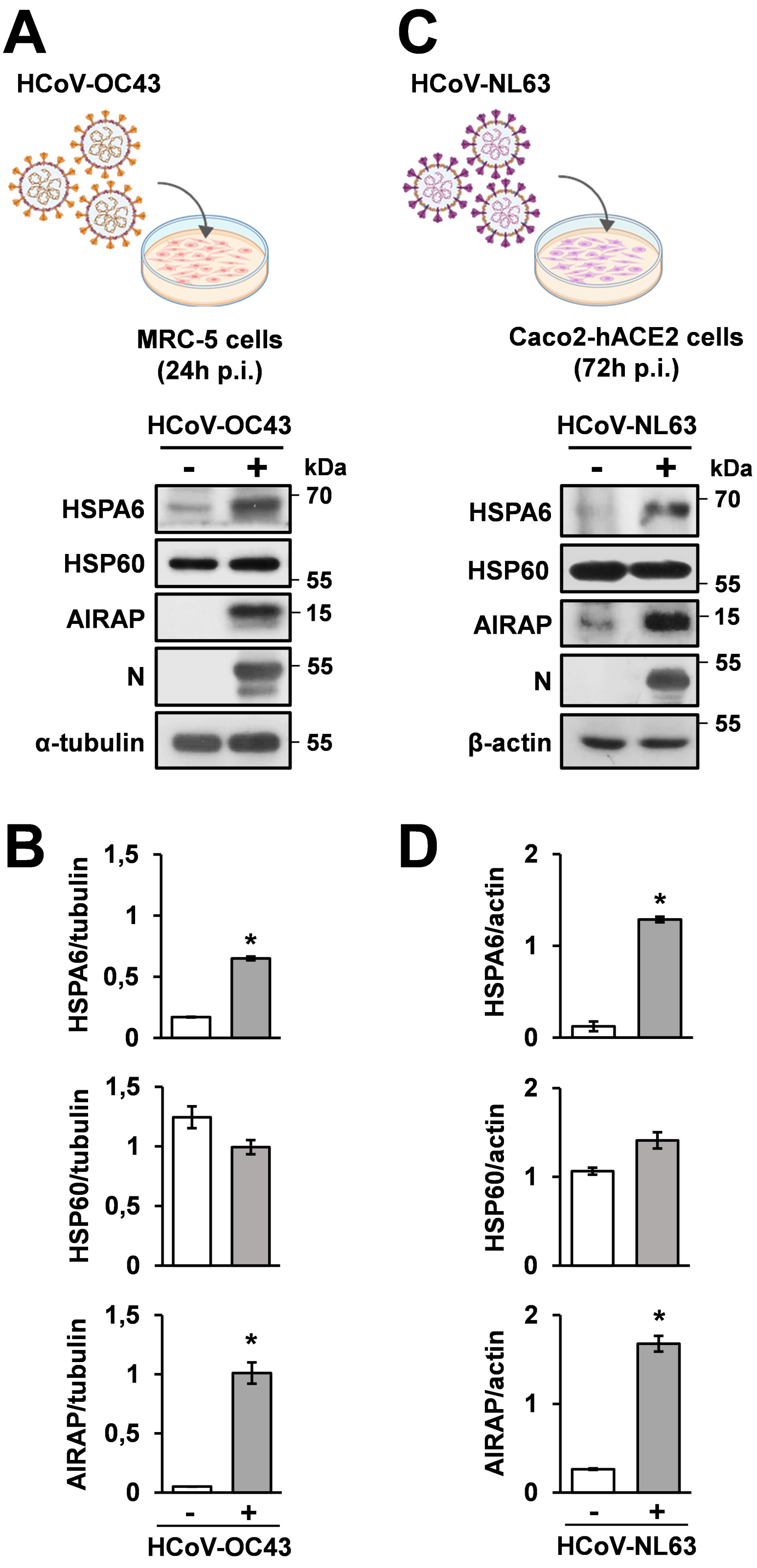
A
HCoV-OC43
MRC-5 cells
(24h p.i.)
HCoV-OC43
- +
kDa
HSPA6
HSP60
AIRAP
N
α-tubulin
70
55
15
55
55
C
HCoV-NL63
Caco2-hACE2 cells
(72h p.i.)
HCoV-NL63
- +
kDa
HSPA6
HSP60
AIRAP
N
β-actin
70
55
15
55
55
B
HSPA6/tubulin
HSP60/tubulin
AIRAP/tubulin
1,5
1
0,5
0
*
HCoV-OC43
D
HSPA6/actin
HSP60/actin
AIRAP/actin
2
1
0
*
HCoV-NL63

Figure S4

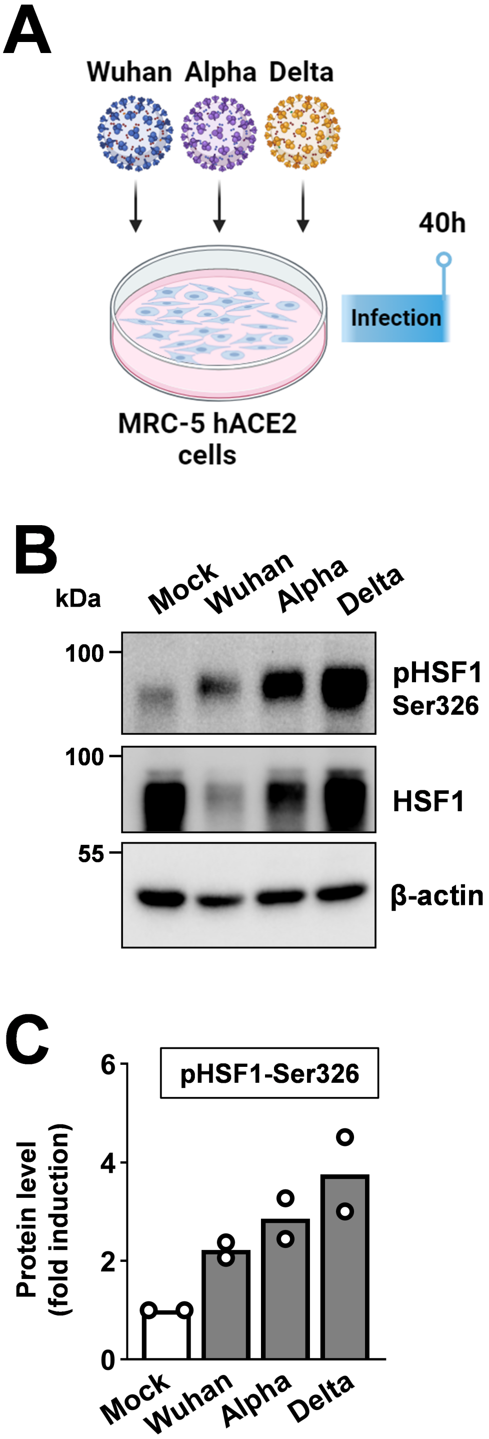
A
Wuhan
Alpha
Delta
40h
Infection
MRC-5 hACE2
cells
B
kDa
Mock
Wuhan
Alpha
Delta
100
pHSF1
Ser326
100
HSF1
55
β-actin
C
pHSF1-Ser326
Protein level
(fold induction)
6
4
2
0
Mock
Wuhan
Alpha
Delta

Figure S5

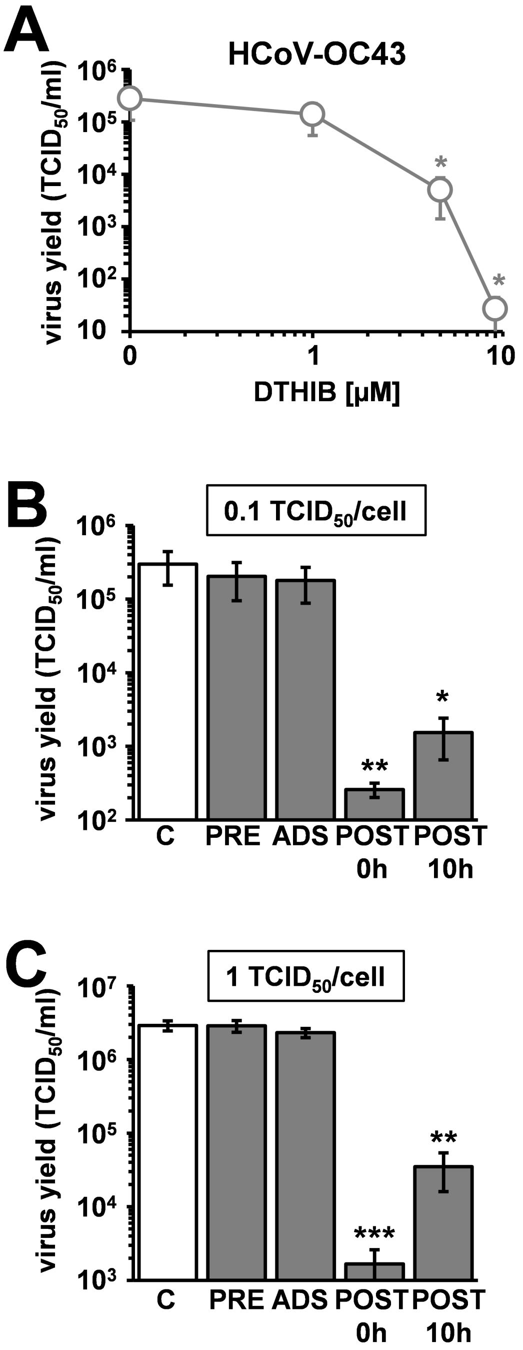
A
HCoV-OC43
virus yield (TCID50/ml)
10⁶
10⁵
10⁴
10³
10²
10
0
1
10
DTHIB [µM]
*
*
B
0.1 TCID50/cell
virus yield (TCID50/ml)
10⁶
10⁵
10⁴
10³
10²
**
*
C
PRE
ADS
POST 0h
POST 10h
C
1 TCID50/cell
virus yield (TCID50/ml)
10⁷
10⁶
10⁵
10⁴
10³
***
**
C
PRE
ADS
POST 0h
POST 10h

Figure S6
